# Nano-iron-modified pomegranate peel powder for rapid and recyclable removal of acetaminophen

**DOI:** 10.1038/s41598-026-48146-6

**Published:** 2026-04-21

**Authors:** Nabel Kalel Asmel, Fadia A. Sulaiman, Abeer I. Alwared, Noor A. Mohammed, Sivarama Krishna Lakkaboyana, Abdullahi Muhammad, Reddi Mohan Naidu Kalla, Ahmad Ansari

**Affiliations:** 1 Building and Construction Technology Engineering, Northern Technical University, Mosul, 41002 Iraq; 2 Department of Geomatics Techniques Engineering, College of Technical Engineering/Mosul, Northern Technical University, Mosul, 41001 Iraq; 3 Department of Environmental Engineering, University of Baghdad, Baghdad, 10071 Iraq; 4 Department of Chemistry, Vel Tech Rangarajan Dr. Sagunthala R&D Institute of Science and Technology, Chennai, 600062 Tamil Nadu India; 5 Department of Fiber System Engineering, Yeungnam University, Gyeongsan, 38541 Republic of Korea; 6 Department of Water Resources and Environmental Engineering, Helmand Higher Education Institute, Lashkar Gah City, Helmand Afghanistan

**Keywords:** Acetaminophen, Pomegranate peels, Nano iron, Adsorption Isotherms, Thermodynamics, Chemistry, Environmental sciences, Materials science, Nanoscience and technology

## Abstract

Pharmaceuticals such as acetaminophen are increasingly detected in the aquatic environment and represent a persistent risk to ecosystems and human health. This study reports the fabrication of nano-iron-loaded pomegranate peel powder (NIL-PP)and its application as a low-cost, magnetically separable adsorbent for the removal of acetaminophen (ACT) from aqueous solutions. Successful loading of nanoscale iron species onto the bio-char matrix was confirmed by SEM-EDS, XRD (average crystallite size is 14 nm), FTIR, BET, TGA, and VSM (18.5 emu/g), indicating a heterogeneous, porous, and magnetically responsive adsorbent. Batch adsorption results indicated that the kinetic data are best described by the pseudo-second-order model, while the equilibrium data fit well with the Freundlich isotherm, suggesting that the adsorption process is predominantly governed by chemisorption occurring as multilayer coverage on energetically heterogeneous surface sites. The optimal removal was observed at an adsorbent dosage of 1 g/L, near neutral pH, an initial ACT concentration of 15 mg/L. The removal rate of ACT exceeded 95.29% within 180 min. Thermodynamic analysis indicates the adsorption is exothermic (ΔH° ≈ −47.13 kJ/mol) and proceeds with overall spontaneous behaviour under test conditions. The NIL-PP material retained a substantial fraction of its capacity (64.33%), even after four consecutive cycles and can be readily separated from water by magnetic collection. This result from this work emphasize the potential of NIL-PP as a suitable adsorbent to be used on a large scale for the environmental remediation of pharmaceutical pollutants in an environmentally sustainable and economically viable manner.

## Introduction

Pharmaceutical contaminants have gained global environmental prominence because of their large-scale manufacture, chemical diversity, widespread use, and environmental persistence at trace-level concentrations in aquatic environments^1,2^. They often gain entry to wastewater systems through excretion and inappropriate disposal as well as industrial discharge, making their way to wastewater treatment plants^3,4^. Pharmaceutical manufacturing, healthcare settings (including hospitals, clinics, and long-term care facilities), and poor disposal of pharmaceutical wastes are the major sources of pharmaceutical pollution^5^.

Acetaminophen (ACT), also known as paracetamol [N-acetyl-p-aminophenol], is a widely used analgesic and antipyretic pharmaceutical for the management of pain and fever. Owing to its extensive consumption and incomplete removal by conventional wastewater treatment processes, acetaminophen has been frequently detected as an emerging contaminant in aquatic environments^6^. Concerns have grown due to its persistence in water bodies and possible negative effects on the environment and public health. The current study suggests an environmentally safe, affordable, and sustainable adsorbent made from agricultural waste as a solution to these problems. Under realistic operating conditions, the synthesized nano-iron ion-enriched pomegranate peel exhibits promising adsorption performance, providing a competitive substitute for commercially available adsorbents. The outcomes of this work contribute to the development of sustainable water treatment strategies and provide a scientific foundation for the design of metal-modified biosorbents aimed at mitigating pharmaceutical pollution in aquatic systems.

In fact, between 58 and 68% ingested amounts of ACT are eliminated unchanged, which further enhances its persistence in aquatic environments^7^ ACT is associated with hepatotoxicity, GI ailments, and liver necrosis in the setting of overdose, even though it is relatively safe at clinically relevant doses. What is alarming is that these endocrine-disruptive effects can take place at concentrations that are even below therapeutic ranges (i.e., doses that cause testosterone suppression)^8^. As the risk of environmental threats increases due to pharmaceutical contaminants, more eco-efficient, affordable, and sustainable water treatment technologies have gained attention^9^. Although utilizing a proven treatment method, traditional treatment approaches are associated with high operational costs, elaborate processing steps, and the production of secondary pollutants^10^.On the other hand, adsorption has become a preferred alternative due to its simplicity, affordability, and versatility^11^.

The adsorption is a surface phenomenon that involves physical and/or chemical interactions between adsorbate molecules present in the gas or liquid phase and the surface of a solid adsorbent. The range of application of this process is typically for the removal of pollutants in the ng/L to mg/L concentration range^12^. Even though activated carbon is the most common adsorbent, it is too expensive for practical application. This sparked an interest in low-cost renewable adsorbents synthesized from agricultural and industrial wastes^13^.

Due to their abundance and low economic value, agricultural waste peels that are rich in lignocellulosic biomass have garnered increased interest for use as sustainable adsorbent precursors. Among these are used tea leaves, pineapple peels^14^, and pistachio shells^15^. Pomegranate (Punica granatum) peel (PP) is a juicing waste item, with ~ 10–15% of th original weight of the fruit^16^ normally discarded. Although a lot of attention has been drawn to PP for its removal of heavy metals^17^.Dyes^18^ and antibiotics^19^ these works are of PP in a dried, unmodified state.

In recent years, much effort has been made on the incorporation of iron-based materials (IBMs) into inorganic matrices for water treatment as they exhibit high surface area, tunable surface charge, and acceptable adsorption behaviors)^20^. Specifically, iron oxides have a mesoporous structure, a neutral low value of point of zero charge, and are environmentally friendly. They are also widely applied in catalysis, as a pigment base, and for pollutant adsorption.

To the best of our knowledge, for the first time, this study reports the synthesis and application of nano-iron ion-enriched pomegranate peel biomass as a functional biosorbent for the removal of acetaminophen from aqueous environments. Integrating nanoscale iron species into the lignocellulosic structure of pomegranate peel improves surface functionality, augments the number of active adsorption sites, and enhances interactions with acetaminophen molecules. Previous studies have mainly investigated raw pomegranate peel or iron-based materials separately as adsorbents. In contrast, the present study investigates the synergistic effect achieved by incorporating agricultural biomass with nano-iron species. To better understand the adsorption process and its underlying mechanism, kinetic, isotherm, and thermodynamic analyses were conducted systematically.

In the current study, nano-iron ion-enriched materials (NIIEM) were synthesized from pomegranate peel and evaluated for the removal of ACT from aqueous solutions. The different parameters were systematically studied, such as adsorbent dosage, contact time, particle size, pH, stirring speed, and ACT concentration for the synthesized adsorbent. Sorption kinetics and isotherm models were used to explain the mechanism of adsorption. This work indicates the promise of NIIEM as an efficient, economical, and sustainable adsorbent for water remediation of pharmaceutical pollutants.

## Materials and methods

### Materials

Acetaminophen (ACT) was obtained from the General Company for Drugs Industry, Iraq. It was selected as a contamination model with a purity of 99% without additional purification. The ACT chemical structure and properties are tabulated in Table 1. The applied iron was present in the form of iron ions (Fe³⁺) incorporated within a nano-iron ion-exchange material (NIIEM, Ecomel 53Nj). Ecomel 53Nj is a proprietary iron-based material supplied by Kobe Steel Ltd. (Japan) at no cost and was used as received without further purification; therefore, it does not possess a single defined chemical formula. According to the manufacturer, iron is present as ionically bound Fe³⁺ species immobilized within a nanostructured matrix. Fresh pomegranates were gathered at a local market’s fresh juice stand, and deionized water was utilized to make solutions for all of the experiments. 0.1 M NaOH and 0.1 M HCl solutions were utilized to modify the sample pH. Every chemical utilized in this investigation was of the caliber of an analytical reagent. The acetaminophen concentration were measured using UV-Vis spectrophotometer at 243 nm.

**Table 1:** ACT Physicochemical properties^21^.

Chemical structure	
Type of drug	Non-steroidal anti-inflammatory drug
Symbol	ACT
Molecular formula	C8H9NO2
Molecular weight, g/mol	151.163
PKa	9.38
Maximum absorption wavelength, nm	243

### The stock solution

ACT powder was added and dissolved in distilled water to prepare the stock solution (1000 mg/L), which was subsequently diluted with distilled water to achieve the required concentrations. Hydrochloric acid (HCl) and sodium hydroxide (NaOH) solutions were used to modify pH levels.

### Preparation and activation of pomegranate peel (PP)

**Fig. 1:**
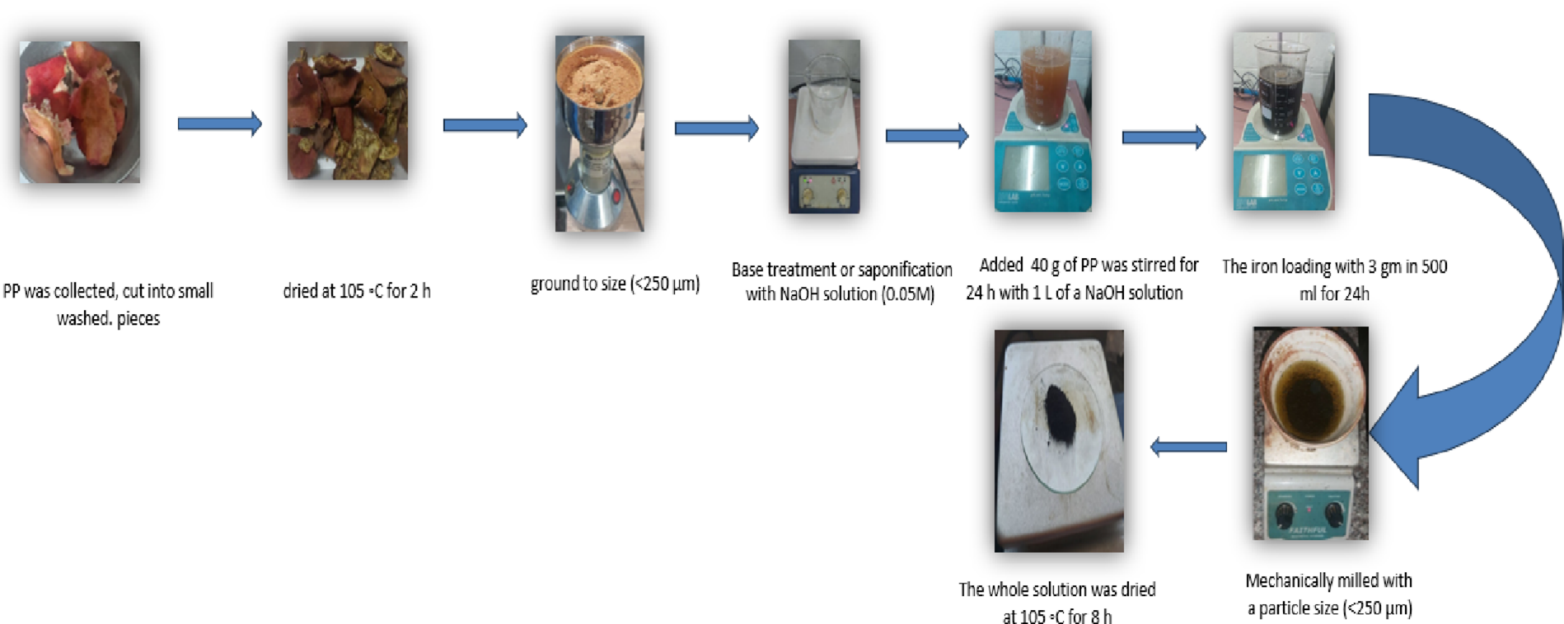
Experimental procedure for NIL-PP preparation.

The PP was activated trough a nano-iron loading method similar to that reported method^22^ Fig. 1 shows a general schematic diagram of the different synthesis steps demonstrated for NIL-PP.

1. First, pomegranate peels (PP) were accumulated, cut into small pieces, and thoroughly splashed with double distilled water a number of times until the rinse water became clear. The material was then oven-dried at 105 °C for 2 h and subsequently ground to the desire particle size (< 250 μm).
2. The base treatment, also known as saponification, was the initial stage in the activation process. 40 g of PP were agitated for 24 h at room temperature with 1 L of 0.05 M NaOH solution. After that, the washing solution was carefully cleaned with distilled water until its pH reached neutral.
3. The second phase, iron loading, was carried out by stirring the saponified PP with 500 ml of a 0.25 M iron solution at room temperature for 24 h.
4. Lastly, the Nano iron-loaded PP (NIL-PP) was oven-dried for 8 h at 105 °C after being thoroughly cleaned with distilled water once again.
5. Before being used in the adsorption tests, it was mechanically ground to the required particle size (< 250 μm) using a planetary ball mill.

### Adsorption procedure

#### Batch adsorption experiments

Batch adsorption experiments were performed in a temperature-controlled shaking water bath (Model 89032-224) maintained at 20 ± 2 °C with a constant agitation speed of 250 rpm. A predetermined mass of NIL-PP was added to a fixed volume of acetaminophen (ACT) solution in sealed Erlenmeyer flasks and agitated to ensure homogeneous suspension and effective solid–liquid interaction.

After the designated contact time, the adsorbent was magnetically separated using an external magnet. The residual ACT concentration was determined by UV–Vis spectrophotometry at λ = 243 nm. Calibration curves were established prior to analysis (R² > 0.995). Blank experiments using deionized water were conducted under identical conditions to eliminate background interference.

All experiments were performed in triplicate, and results are presented as mean ± standard deviation (SD). Error bars in all graphical representations correspond to ± SD. The standard operating conditions were: initial ACT concentration: 15 mg/L, adsorbent dosage: 1 g/L, solution pH: 5.0, temperature: 20 °C, particle size: ~2 μm, agitation speed: 250 rpm, The amount of ACT adsorbed at time t and at equilibrium was determined using the mass balance relationship:

1 $$\:q=\frac{{c}_{i}-{c}_{f}}{w}\times\:v$$

where q (mg/g) represents the adsorption capacity; C_i_ and Cf (mg/L) denote the initial and final ACT concentrations, respectively; V (L) is the solution volume; and W (g) is the dry mass of adsorbent. This calculation is based on the concentration difference between the initial and residual ACT in solution.s.

#### Kinetic studies

Adsorption kinetics were investigated over a contact time range of 5–180 min under constant experimental conditions. Samples were withdrawn at predetermined intervals, immediately separated magnetically, and analyzed. The experimental data were evaluated using the non-linear forms of the following kinetic models: Pseudo-first-order (PFO), Pseudo-second-order (PSO), Elovich model, and Intraparticle diffusion (Weber–Morris) model. Model parameters were estimated using non-linear regression analysis (Microsoft Excel Solver, GRG nonlinear algorithm) by minimizing the sum of squared errors between experimental and predicted values.

To ensure rigorous model discrimination, kinetic fitting was assessed using multiple statistical criteria: Coefficient of determination (R²), Standard error (SE), Normalized standard deviation (NSD), Average relative error (ARE). The most appropriate kinetic model was selected based on the highest R² and the lowest associated error values, ensuring statistical robustness and minimizing overreliance on linear correlation.

#### Equilibrium isotherm studies

Equilibrium isotherm experiments were conducted by varying the initial ACT concentration from 5 to 100 mg L⁻¹ under controlled conditions (adsorbent dose = 1 g L⁻¹, pH = 5.0, agitation speed = 250 rpm, contact time = 180 min). To investigate the temperature dependence of adsorption and enable thermodynamic evaluation, isotherm experiments were additionally performed at 20, 30, 40, 50, and 60 °C using a temperature-controlled shaking system.

The equilibrium data were analyzed using the non-linear forms of: Langmuir isotherm model, Freundlich isotherm model, and Langmuir–Freundlich isotherm model.The dimensionless separation factor (R_L_) was calculated from the Langmuir constant to evaluate adsorption favorability.

Isotherm model performance was assessed using: Coefficient of determination (R²), Standard error (SE), Root mean square error (RMSE), Chi-square statistic (χ²), Linear regression coefficient (R²) for comparison with linearized forms. Although linear regression coefficients were reported, model selection was primarily based on non-linear fitting results to avoid potential bias introduced by equation linearization.

#### Thermodynamic analysis

Thermodynamic parameters were determined from adsorption experiments conducted at 20–60 °C under constant experimental conditions. The equilibrium constant was calculated from adsorption data, and standard Gibbs free energy (ΔG°), enthalpy (ΔH°), and entropy (ΔS°) changes were derived from temperature-dependent equilibrium relationships.

The statistical reliability of thermodynamic calculations was verified through linear regression analysis, with corresponding R² values and standard errors reported to confirm consistency and robustness. All adsorption experiments were conducted in triplicate, and the results are reported as mean ± standard deviation.

### Regeneration experiments

Regeneration of NIL-PP was carried out by desorbing the adsorbed acetaminophen using an ethanol–water solution (70:30, v/v) under mild stirring for 60 min at ambient temperature. After desorption, the adsorbent was washed repeatedly with deionized water until neutral pH was reached, dried, and then reused in subsequent adsorption cycles.

## Results and discussion

### Characterization analysis results

#### Fourier transform infrared spectroscopy (FTIR)

**Fig. 2:**
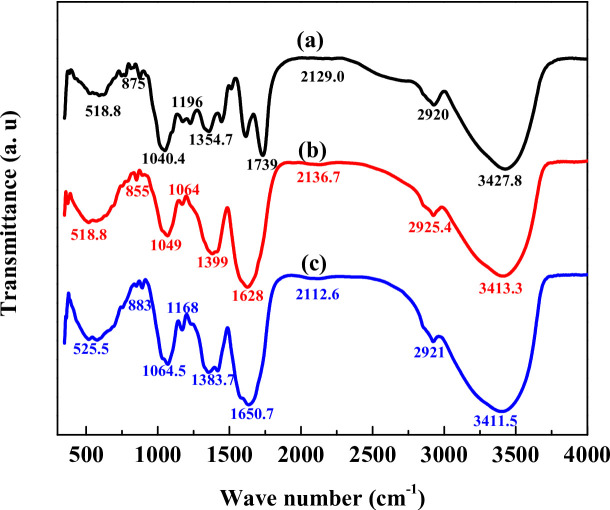
FTIR analysis: (**a**) pure PP, (**b**) NIL-PP before use, (**c**) NIL-PP after use.

The Fourier transform infrared (FTIR) spectra of pomegranate peel powder (PP), nano-iron-loaded pomegranate peel (NIL-PP) before adsorption, and NIL-PP after acetaminophen (ACT) adsorption are presented in Fig. 2 to identify surface functional groups and their involvement in the adsorption process. The FTIR spectrum of raw PP (black line) exhibits prominent bands at 3413.3 cm⁻¹, 2925.4 cm⁻¹, and 1628 cm⁻¹, which are attributed to O–H stretching vibrations of hydroxyl groups^23^, C–H stretching of aliphatic chains, and C = O stretching of carboxyl groups, respectively^24^.Bands observed in the range of 1049–1399 cm⁻¹ correspond to C–O and C–C vibrations associated with polyphenolic and cellulose-like structures. Weak bands at 518.8 cm⁻¹ and 855 cm⁻¹ are assigned to bending vibrations of aromatic rings or inorganic components^25^.

Following iron loading (NIL-PP before adsorption), noticeable spectral changes are observed. The broad O–H stretching band slightly shifts from 3413.3 to 3411.5 cm⁻¹, indicating possible hydrogen bonding interactions or coordination between iron species and surface hydroxyl groups^26^. Enhanced bands at 1168, 1065, and 1335.7 cm⁻¹ suggest interactions between iron and oxygen-containing functional groups, likely forming C–O–Fe or Fe–OH linkages. In addition, the shift of the band from 1628 to 1658.7 cm⁻¹ implies modification of the carboxyl group environment following iron incorporation^27^. A weak band appearing at approximately 2112.6 cm⁻¹ may be associated with the formation of new surface complexes after iron loading.

After ACT adsorption (red spectrum), further spectral changes are evident, confirming successful interaction between ACT molecules and the NIL-PP surface. The O–H band at 3411.5 cm⁻¹ persists with altered intensity, suggesting hydrogen bonding between ACT and surface hydroxyl groups^28^. Changes in the region of 600–1700 cm⁻¹, including increased intensity and band broadening, are consistent with contributions from the aromatic ring and amide functional groups of ACT. Alterations in the fingerprint region (400–1500 cm⁻¹), particularly band shifting and broadening, further indicate surface modification and adsorption of ACT molecules on NIL-PP^29^.

Overall, FTIR analysis confirms the presence of reactive functional groups on PP, successful iron incorporation, and subsequent adsorption of acetaminophen. The observed spectral shifts suggest that adsorption occurs primarily through hydrogen bonding and possible surface complexation, while electrostatic interactions may contribute only secondarily under the studied pH conditions^22^. The involvement of Fe–acetaminophen coordination and π–π interactions is proposed based on indirect experimental evidence and literature precedent; however, direct confirmation using advanced spectroscopic techniques (e.g., XPS or solid-state NMR) was beyond the scope of this study^29^.

#### Scanning electron microscope (SEM)

**Fig. 3:**
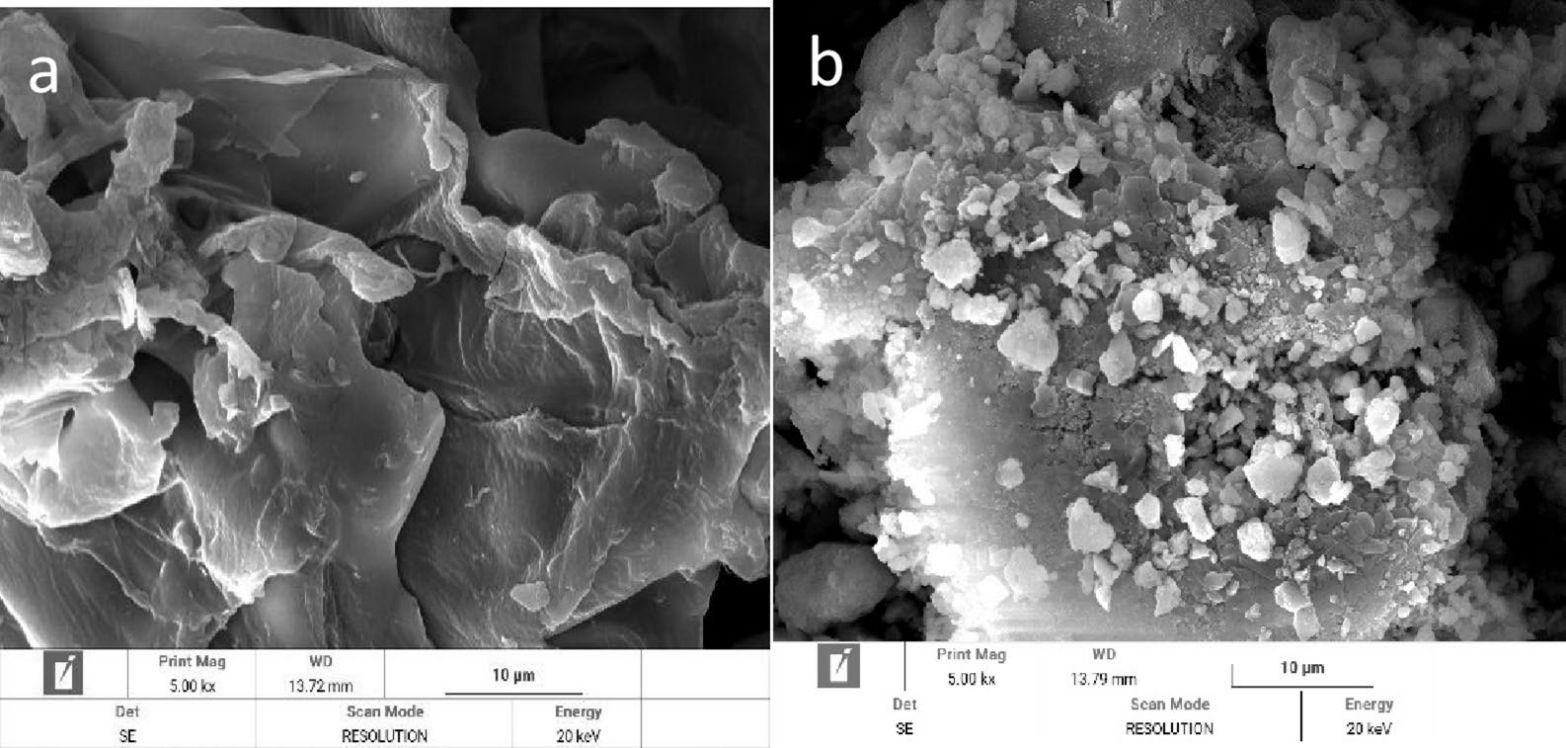
SEM analysis: (**a**) pure PP, (**b**) NIL-PP before use.

Scanning electron microscope (SEM) micrographs Fig. 3 of (a) pomegranate peel powder (PP), and (b) nano-iron particles supported by PP Nano-Iron loaded PP (NIL-PP) Before Usage (Fig. 3b).

In this image, the exterior pomegranate peel powder is densely filled with granular structures, which also suggests successful deposition of nano iron particles. The distribution is rather homogeneous, while the smaller bright points (nano-iron) stand out on the surface.

Significantly, the nano-iron particles were shown to aggregate or react after use, which resulted in larger and more irregular clumps. The surface is not completely uniformly covered, and some structural modifications to the peel powder may have occurred due to the reaction. Such a comparison very well displays the morphology of the material before (after nano-iron loading) and after (post-application) the exposure^30^.

#### Elemental composition analysis (EDS)

**Fig. 4:**
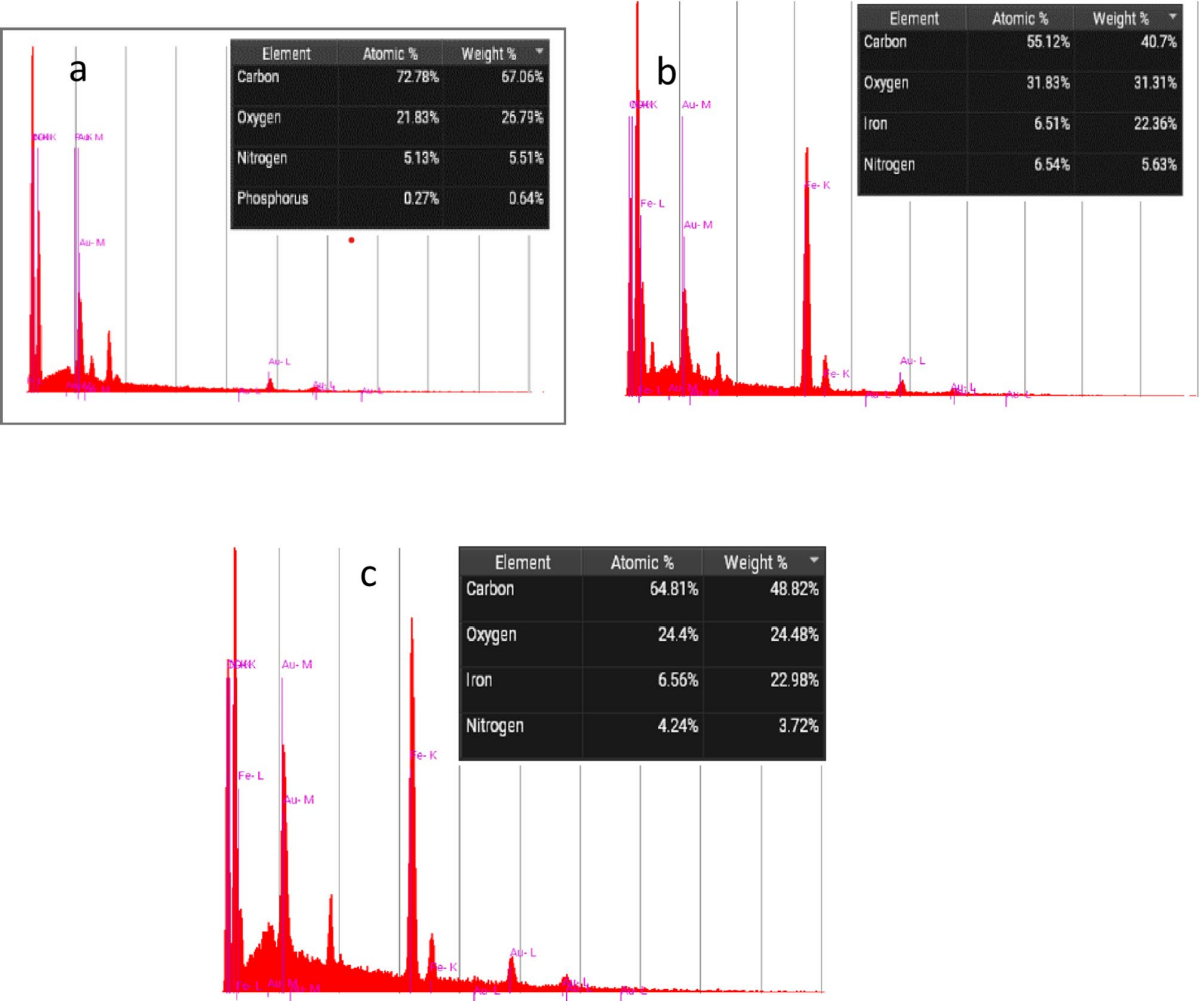
EDS analysis: (**a**) pure PP, (**b**) NIL-PP before use, (**c**) NIL-PP after use.

Figure 4 displays the EDS spectra of the samples of (a) pure PP, (b) NIL-PP before use, and (c) NIL-PP after adsorption. This method is a confirmation of the elemental analysis of materials which is also in support of morphological and functional properties determination via SEM and FTIR.

Spectrum (a) depicts the EDS profile of pure PP, where strong signals of carbon (c) and oxygen (O) are observed, originating primarily from organic biomass rich in cellulose, hemicellulose, lignin, as well as polyphenolic compounds. The low levels of potassium (K) and residual amounts of additional elements, including phosphorus (P) and calcium (Ca), correspond to the mineral content found in most natural source plant-based materials The lack of iron (Fe) indicates the absence of any iron-informed modification at this stage.

Spectrum (b) refers to NIL-PP before applying. In this regard, high and China peaks of Fe are seen, verifying the deposition of nano-iron particles on the PP substrate. These iron peaks, especially Fe-K and Fe-L, give a direct indication of the existence of metallic iron. The appearance of C and O shows that the organic matrix of PP has not been excluded.

In spectrum (c), characteristic Fe signals still appear, confirming the stability of the Fe layer of nano-iron even after the acetaminophen molecules had adsorbed (the acetaminophen was added for 12 h before the spectrum was taken). Only small variations in the elemental intensities were found wherein the oxygen content increased, which indicates an interaction of the NIL-PP surface with the acetaminophen by possible oxidation or hydrogen bonding mechanisms. The presence of Fe peaks also indicates that iron is not leached during adsorption, thus favoring the reusability of the material^31^.

EDS analysis to prove PP has been successfully functionalized with nano-iron and that the resulting composite maintains its chemical stability after adsorption. The combined results of the SEM and FTIR analyses also provide further evidence that NIL-PP has potential as a stable and reactive adsorbent for the removal of pharmaceutical contaminants from aqueous solutions.

#### X-ray diffractometry (XRD)

**Fig. 5:**
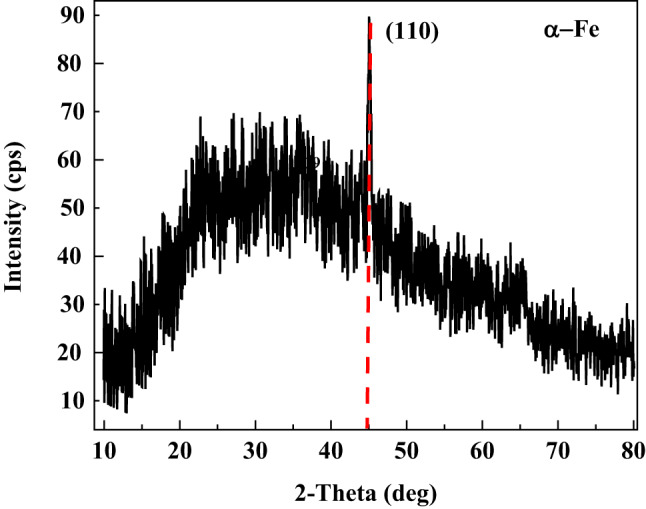
XRD pattern for NIL-PP.

Figure 5 presents the X-ray diffraction (XRD) pattern of nano-iron-loaded pomegranate peel powder (NIL-PP). The diffractogram exhibits a broad diffuse halo in the 15–30° (2θ) region, characteristic of amorphous lignocellulosic materials. This feature confirms that the fundamental structure of the pomegranate peel matrix remains predominantly amorphous following iron incorporation.

Superimposed on this amorphous background, a distinct diffraction peak is observed at approximately 44.7° (2θ). This reflection is indexed to the (110) crystallographic plane of body-centered cubic (BCC) α-Fe (PDF No. 06–0696), indicating the presence of zero-valent iron (Fe⁰) as the principal crystalline phase^32^. A weak feature is observed near 65°; however, due to its low intensity and limited resolution, it cannot be conclusively assigned to a specific crystallographic plane. The absence of additional well-defined reflections associated with iron oxides suggests that metallic iron is the dominant detectable phase under the applied measurement conditions.

The coexistence of a sharp metallic iron reflection and the broad amorphous halo confirms the successful immobilization of crystalline iron domains within the biomass matrix without altering its structural integrity^33^. This hybrid architecture integrates a nanocrystalline iron phase, providing reactive redox-active sites, with an amorphous biopolymeric scaffold rich in surface functional groups, thereby generating a composite structure favorable for adsorption and redox-assisted contaminant removal^34,35^.

The average crystallite size of the iron phase was estimated using the Debye–Scherrer equation (Eq. 2):

2 $$\:D\frac{k\lambda\:}{\beta\:cos\theta\:}$$

where D represents the crystallite size (nm), K is the Scherrer constant (0.94), λ is the X-ray wavelength (Cu–Kα, 0.15418 nm), β is the full width at half maximum (FWHM) of the selected diffraction peak after instrumental correction (in radians), and θ is the Bragg diffraction angle. Based on the FWHM of the principal (110) reflection, the calculated average crystallite size was 14.044 nm, confirming the nanocrystalline nature of the immobilized iron phase.

#### Vibrating sample magnetometer (VSM) analysis

**Fig. 6:**
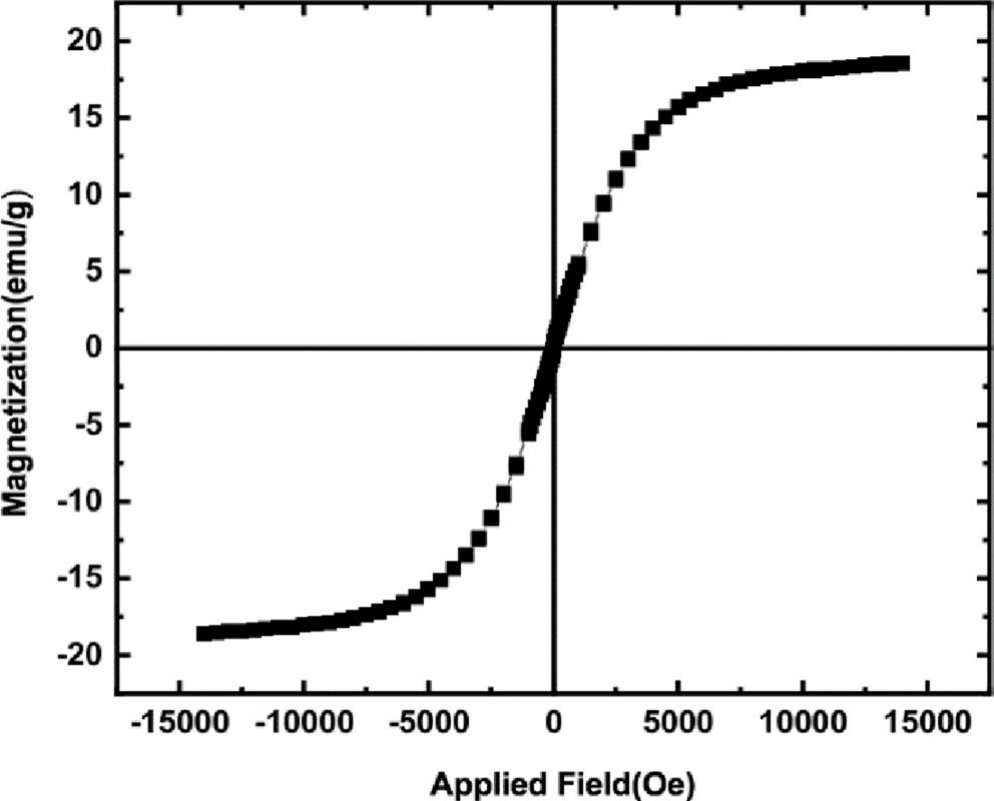
VSM analysis of NIL-PP.

The magnetic properties of iron-loaded pomegranate peel powder (NIL-PP) were evaluated using vibrating sample magnetometry (VSM) to assess its suitability for magnetic separation after adsorption. As shown in Fig. 6, the magnetization curve exhibits a typical S-shaped hysteresis loop with symmetry about the origin, which is characteristic of quasi-superparamagnetic behavior. The steep slope near the origin, together with the negligible coercivity (Hc) and remanent magnetization (Mr), indicates rapid realignment of magnetic domains under an external magnetic field and near-zero residual magnetization upon field removal.

The saturation magnetization (Ms) of NIL-PP was determined to be 18.5 emu g⁻¹, confirming the successful incorporation of nano-iron particles within the pomegranate peel matrix. Although this Ms value is lower than that of bare magnetite nanoparticles, it remains sufficient for rapid and efficient magnetic recovery from aqueous media. Importantly, the low Hc and Mr values are advantageous for adsorption–desorption cycles, as they minimize particle aggregation and facilitate repeated recovery using an external magnet.

For comparison, activated carbon incorporating protected Ni nanoparticles exhibited Ms values in the range of 20–30 emu g⁻¹, enabling effective magnetic separation after adsorption processes^36^. Similarly, magnetized activated carbon derived from pomegranate husk showed Ms values of approximately 15–25 emu g⁻¹, which were attributed to dilution of the magnetic phase within the carbon matrix while maintaining good recoverability recoverability^31,37^. In contrast, significantly higher Ms values have been reported for dense magnetic nanostructures, such as 66.5 emu/g for bare Fe₃O₄ nanoparticles and approximately 88 emu g⁻¹ for Fe-based nanocomposites characterized using VSM or SQUID techniques^38^. However, such systems are often less favorable for adsorption applications due to reduced surface functionality and increased aggregation tendencies.

The moderate Ms value of NIL-PP is therefore consistent with other biomass-derived magnetic carbon adsorbents and reflects partial shielding of iron nanoparticles within the porous matrix. This level of magnetization provides an optimal balance between magnetic separability and adsorption efficiency, which is essential for adsorbent recovery, regeneration, and repeated reuse. Similar observations have been reported for magnetic nanocomposites applied to antibiotic and pharmaceutical removal, where moderate magnetization ensured recyclability without compromising adsorption performance^39,40^. Overall, the VSM results confirm that NIL-PP possesses adequate magnetic responsiveness for practical post-adsorption separation while preserving the surface properties required for effective ACT adsorption.

#### Surface area of the NIL-PP

**Fig. 7:**
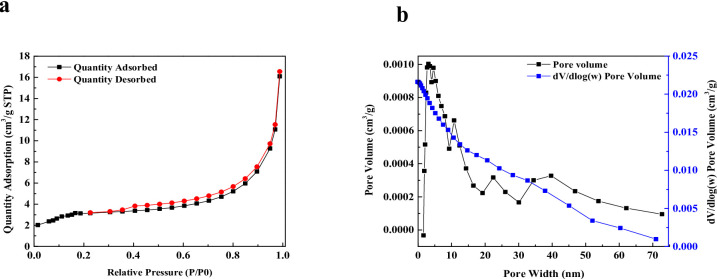
(**a**) N_2_ adsorption/desorption isotherms (**b**) Pore size distribution and pore volume of raw NIL-PP.

The N₂ adsorption–desorption isotherm of nano-iron-loaded pomegranate peel powder (NIL-PP) is presented in Fig. 7a. The isotherm exhibits a typical Type IV profile with an H3 hysteresis loop, according to the IUPAC classification, confirming the predominance of a mesoporous structure. Type IV isotherms are characteristic of materials containing pores within the 2–50 nm range. The absence of a distinct adsorption plateau at high relative pressures and the continuous increase in nitrogen uptake at P/P₀ > 0.8 indicate capillary condensation occurring in slit-shaped mesopores formed by the aggregation of plate-like particles rather than uniform cylindrical pores^41^.

In the low relative pressure region (P/P₀ < 0.2), the gradual increase in adsorption suggests limited microporosity and/or strong adsorbate–surface interactions^42^. The H3 hysteresis loop further supports the presence of non-rigid slit-like pores and heterogeneous pore geometry, which are commonly observed in biomass-derived carbonaceous materials and nanocomposites. The asymmetric nature of the loop reflects structural disorder and pore connectivity variations within the modified matrix.

The pore size distribution shown in Fig. 7b confirms that the majority of pores are centered within the mesoporous region, with an average pore diameter of 8.51 nm, as summarized in Table 2. The total pore volume was determined to be 0.0249 cm^3^/g, with a BET surface area of 11.70 m²/g and a Langmuir surface area of 15.19 m²/g. Although the BET surface area is lower than that of commercial activated carbons, the mesoporous architecture and accessible pore volume provide sufficient diffusion pathways for acetaminophen (ACT) molecules^43^.

Importantly, the pore diameter (8.51 nm) is significantly larger than the molecular dimensions of ACT, facilitating unobstructed transport toward active adsorption sites. Therefore, adsorption is likely governed primarily by surface functionality and site-specific interactions (e.g., hydrogen bonding, π–π interactions, and coordination with iron-modified sites identified by FTIR) rather than by surface area alone. This interpretation is consistent with the kinetic and isotherm analyses discussed in subsequent sections.

The N₂ adsorption–desorption analysis demonstrates that nano-iron incorporation does not compromise the intrinsic pore architecture of the pomegranate peel matrix. Instead, the composite preserves a stable mesoporous framework with accessible diffusion channels. The synergistic coexistence of preserved mesoporosity and iron-induced active sites enhances surface-mediated interactions and overall adsorption efficiency. Collectively, these findings establish NIL-PP as a structurally stable and functionally active mesoporous adsorbent with promising potential for environmental remediation applications.

**Table 2:** Textural properties of NIL-PP obtained from N₂ adsorption–desorption analysis.

Specification	Unit	Value
BET surface area	m²/g	11.70
Langmuir surface area	m²/g	15.19
BJH surface area	m²/g	4.56
Total pore volume	cm³/g	0.0249
Average pore diameter	nm	8.51
BET surface area	m²/g	11.70

#### Thermogravimetric (TG) and derivative thermogravimetric (DTG) analysis of NIL-PP

**Fig. 8:**
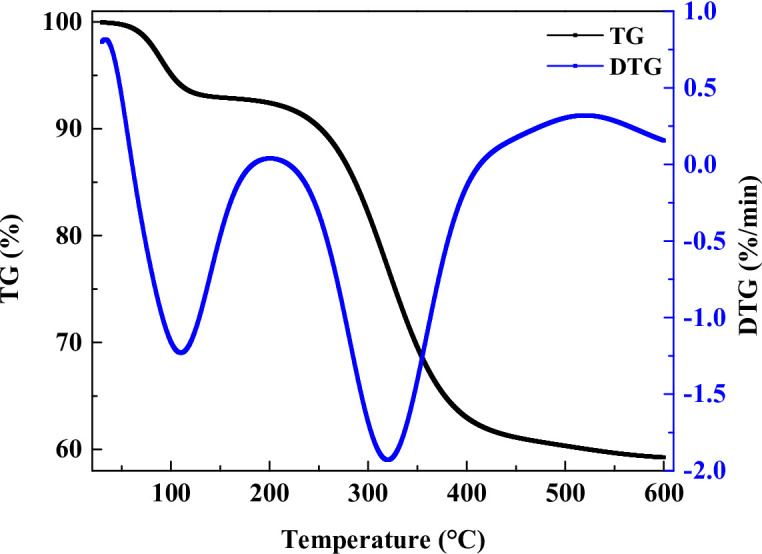
TG and TGA analysis of NIL-PP.

Figure 8 illustrates the TG and DTG profiles of the NIL-PP composite recorded from room temperature up to 600 °C. The thermogram reveals a multi-step degradation process governed by sequential removal of volatile species, decomposition of the organic biomass matrix, and stabilization of the inorganic fraction.The TG curve shows an initial weight loss of approximately 8.0% in the temperature range of 39–174 °C. This stage is attributed to the desorption of physically adsorbed moisture and low-molecular-weight volatile compounds retained within the porous pomegranate peel structure. The relatively small magnitude of this loss confirms low free-water content and good pre-treatment of the material^44^.

The principal mass loss occurs between 176 and 593 °C and accounts for the remaining ~ 32% of weight reduction^33^. When combined with the initial stage, the total mass loss reaches approximately 39–40%, consistent with the final residual mass of ~ 60.8% at 600 °C. This major degradation stage corresponds to the thermal decomposition of the organic constituents of the pomegranate peel matrix, including hemicellulose, cellulose, lignin fragments, and polyphenolic compounds.From a mechanistic perspective, this stage involves the cleavage of β-(1→4) glycosidic linkages in cellulose, the depolymerization of hemicellulose side chains, and the fragmentation of C–O–C ether bonds as well as aromatic linkages within lignin. These bond-breaking processes are accompanied by the evolution of carbon monoxide (CO), carbon dioxide (CO₂), and other volatile decomposition products.

The DTG curve exhibits two distinct peaks. The first peak at ~ 105 °C corresponds to rapid moisture evaporation, while the second, more intense peak at ~ 330–335 °C represents the maximum degradation rate of the organic matrix. This temperature is characteristic of cellulose depolymerization and lignocellulosic backbone breakdown.

Above 500 °C, the TG curve approaches a plateau with a residual mass of approximately 60.8%. This high residue cannot be attributed solely to biomass char formation, as pristine lignocellulosic materials typically leave significantly lower ash content. Therefore, the elevated residual fraction strongly indicates the presence of thermally stable inorganic components, primarily iron-based nanoparticles (e.g., iron oxides) embedded within the composite matrix.

During thermal treatment, nano-iron species remain stable and may also promote catalytic carbonization reactions, enhancing char formation through dehydration and aromatization pathways. As a result, the residual mass consists of a carbonaceous framework reinforced by iron oxide nanoparticles.

Overall, the TG/DTG results confirm that NIL-PP undergoes approximately 40% total mass loss, while retaining a substantial inorganic-rich residue. The high residual fraction and controlled degradation profile demonstrate enhanced thermal stability compared to raw biomass, highlighting the structural robustness of the nano-iron-loaded composite.

#### X-ray fluorescence (XRF)

As indicated in Table 3, analysis was performed to ascertain the elemental composition of NIL-PP. The findings showed that at 45.331% of the substance, Fe_2_O_3_ was the main component. Furthermore, the granite structure showed a low K_2_O content of 3.432%. The percentage contents of the other elements with atomic numbers less than 11 are impossible^45^. The difference in elemental percentages between the EDX and XRF results arises from the exclusion of the “O” in the XRF analysis. Nonetheless, both tests showed that the NIL-PP structure had a high Fe content, with almost identical values.

**Table 3:** XRF analysis of NIL-PP.

Elements	NIL-PP)%)
Na_2_O	3.296
MgO	0.181
SiO_2_	0.297
P_2_O_5_	0.318
SO_3_	0.724
Cl	0.779
K_2_O	3.432
CaO	0.806
Cr	0.011
MnO	0.092
Fe_2_O_3_	45.331
Ni	0.023
Cu	0.038
Zn	0.03

### Batch adsorption results

#### Influence of point of zero charge (pH_pzc_)

**Fig. 9:**
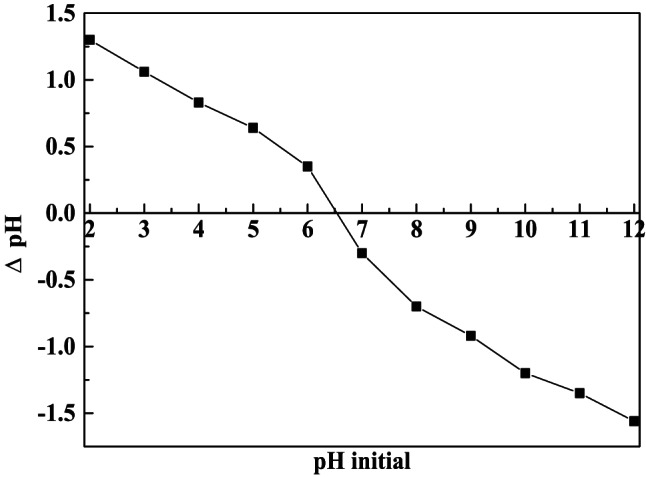
Determination of the Point of Zero Charge (pHpzc) of NIL-PP.

The point of zero charge (pH_pzc_) of the NIL-PP composite was determined by plotting the difference between pH final and pH initial (∆pH = pHfinal – pHinitial) versus the initial pH of the NIL-PP composite, as shown in Fig. 9. The intersection of this curve with the horizontal axis (∆pH = 0) represents the pH_pzc_ or pH at which the adsorbent surface has a net zero charge. Notably, the intersection point of this plot appears to be about pH 6.7, which suggests the pH_pzc_ of NIL-PP. As the pH falls below this value, the protonated to become positively charged, which may result in better adsorption of the anionic species^46^. On the other hand, between pH 6.7 and 10, the surface acquires a negative charge that allows cationic species to preferentially adsorb owing to deprotonation.

The kinetic trend observed, whereby ∆pH is positive at low initial pH and negative at high initial pH, is consistent with that expected for functionalized bio-adsorbents. This finding validates the atmospheric characteristics of the NIL-PP surface, which is essential for maximizing its function in pH-driven adsorption mechanisms like contaminant removal from aqueous media. For the application of the material in environmental remediation, where interaction with charged pollutants is impacted by the surface charge of the adsorbent, the pH_pzc_ is critical.

Figure 9 represents the ∆pH (pH_final_-pH_initial_) versus initial pH curve used to determine the point of zero charge (pH_pzc_) of the NIL-PP adsorbent in the context of acetaminophen (ACT) adsorption. The point at which the curve crosses the horizontal axis (∆pH = 0) corresponds to the pH_pzc_, identified at approximately pH 6.7. This value signifies the pH at which the surface of the NIL-PP carries no net electrical charge^47^.

At pH values below 6.7, the surface of NIL-PP is predominantly protonated, resulting in a net positive surface charge. However, since acetaminophen exists mainly in its neutral molecular form under these conditions, electrostatic interactions are expected to be minimal. Adsorption is therefore primarily governed by hydrogen bonding and specific surface interactions rather than charge-driven attraction^46^. Conversely, at pH values above 6.7, the surface becomes negatively charged due to deprotonation of surface functional groups, which may reduce interaction with acetaminophen depending on its ionization state.

The pHpzc is a critical parameter influencing adsorption behavior, as acetaminophen (pKa ≈ 9.5) exists predominantly in its neutral form under acidic to slightly basic conditions. Therefore, near or just below the pHpzc, adsorption is mainly governed by non-electrostatic interactions such as hydrogen bonding, π–π stacking, and hydrophobic interactions, while electrostatic effects are expected to play only a secondary role and become more relevant only at pH values approaching the ionization range of acetaminophen^48^.

Understanding the surface charge behavior of NIL-PP in relation to pH is essential for optimizing acetaminophen removal from aqueous solutions. This knowledge aids in predicting adsorption mechanisms and tailoring operating conditions for maximum adsorptive performance in pharmaceutical wastewater treatment applications.

#### The impact of pH on the adsorption process

**Fig. 10:**
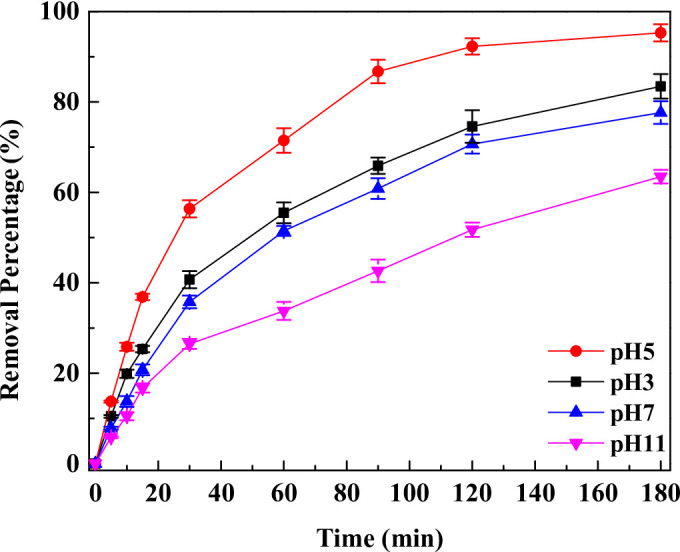
Effect of pH on Acetaminophen (ACT) Adsorption onto NIL-PP under controlled experimental conditions (initial ACT concentration: 15 mg/L; adsorbent dosage: 1 g/L; temperature: 20 °C; and agitation speed: 250 rpm).

The influence of initial solution pH on the ACT removal efficiency of NIL-PP over time is presented in Fig. 10. Adsorption performance as a function of pH is also highly pH-dependent^49^ and the maximum removal was found at pH 5 with an approximate value of 94% after 180 min. At pH 11, the adsorption penalty is very severe, and only 63% removal was achieved, indicating that the removal efficiency decreases at both lower and higher pH values.

The higher adsorption at pH 5 is explained in terms of favorable interactions between the NIL-PP surface and acetaminophen molecules. The pH_pzc_ (∼6.7) shows that the NIL-PP surface is slightly positive at this pH. Due to being mostly in its neutral molecular form at this pH, acetaminophen is more likely to be adsorbed through hydrogen bonding, π-π interactions between the aromatic rings of ACT and NIL-PP, and possible coordination with the iron sites existing on the NIL-PP surface^48^ This pH condition provides an optimal balance in which the surface functional groups of NIL-PP remain readily accessible for non-covalent interactions, while electrostatic effects are minimal due to the largely neutral speciation of acetaminophen. At lower pH values (e.g., pH 3), although the NIL-PP surface becomes more positively charged, competition from excess H⁺ ions for surface functional groups can reduce the availability of active sites for hydrogen bonding and other specific interactions, thereby diminishing ACT adsorption.

Moreover, ACT will most exist in the un-ionized form, reducing static attraction. Alternatively, at elevated pH (11, for example), the NIL-PP surface is negatively charged with ACT species (phenolate form), resulting in a tremendous loss of adsorption efficiency.

The adsorption mechanism of acetaminophen (ACT) onto NIL-PP as a function of pH involves several physicochemical interactions that vary depending on the charge of both the adsorbent and adsorbate. Here is a detailed pH-based mechanism:

1. **pH< pHpzc (Acidic conditions**,** pH < 6.7)**:

The NIL-PP surface is positively charged under pH < 6.7, and acetaminophen speciation is predominantly in molecular (neutral) form due to its pKa ∼ 9.5. So, possible mechanism was proceed^49^.

- **Hydrogen Bonding**: Between -OH and -NH groups of ACT and functional groups (carboxyl, hydroxyl) on NIL-PP.
- **π - π Interactions**: Between the aromatic ring of ACT and phenolic/aromatic structures in the pomegranate peel.
- **Coordination with Iron Sites**: The lone pair electrons of ACT function groups may coordinate with Fe centers.
- **Minimal electrostatic contribution**: Since acetaminophen exists predominantly in a neutral form under the studied pH conditions, electrostatic interactions with the NIL-PP surface are negligible, allowing non-electrostatic interactions (e.g., hydrogen bonding and π–π interactions) to dominate the adsorption process.
  2. **pH ≈ pH**_**pzc**_ **(~6.7)** :
- Similar to acidic pH, but slightly reduced in efficiency due to decreased availability of positively charged sites.
- Adsorption remains favorable due to continued hydrogen bonding and π - π stacking.
  3. **pH > pHpzc (Alkaline Conditions**,** pH > 7)** :
- **Negligible electrostatic effects**: Since acetaminophen remains predominantly neutral under the studied pH conditions, electrostatic repulsion between ACT and the NIL-PP surface is minimal, and adsorption is governed mainly by non-electrostatic interactions^49^.
- **Reduced Hydrogen Bonding**: Deprotonation of ACT reduces hydrogen bonding.
- **π - π and Van der Waals Interactions**: Still possible but insufficient to counteract repulsion.

This pH-dependent behavior indicates that maximum ACT adsorption occurs under mildly acidic conditions, particularly around pH 5, where favorable non-covalent interactions such as hydrogen bonding and π–π interactions are maximized, while electrostatic effects remain minimal.

1. **Simplified Functionalization of NIL-PP (Simplified)**:

Pomegranate peel contains hydroxyl (-OH), carboxyl (-COOH), and phenolic groups. When loaded with iron, Fe species can also act as coordination sites.

NIL-PP surface ≈ -COOH, OH, -Fe-OH.

In aqueous media:

COOH ↔ -COO^-^ + H^+^ (pH dependent).

Fe-OH + H^+^ ↔ Fe-OH_2_^+^ (acidic pH).

Fe-OH + H^+^ ↔ Fe-O^-^ + H^+^ (alkaline pH).

2. **Acetaminophen Structure and Ionization**.

ACT (C_8_H_9_NO_2_) has a hydroxyl and an amide group:

HO-C_6_H_4_-NH-COCH_3_.

At low pH (acidic), it remains neutral:

ACT ↔ ACT⁰ (neutral).

At high pH (alkaline), the phenolic group may deprotonate:

ACT-OH ↔ ACT-O^-^ + H^+^ (pKa ≈ 9.5).

3. ***Adsorption Mechanism Equations*** :
  A. *Acidic pH (pH< pH*_*pzc*_*)*:

- Surface is positively charged (e.g., Fe-OH_2_^+^).
- ACT remains neutral.

Mechanisms: Hydrogen Bonding:

-COOH… HO-C_6_H_4_-NHCOCH_3_ (H-bond between carboxyl and phenol/amide) Coordination with Fe:

Fe-OH_2_^+^ + HO-C_6_H_4−_NH-COCH_3_ → -Fe-O-C_6_H_4_-NH-COCH_3_ + H_2_O.

π - π Interactions:

Aromatic groups in NIL-PP ≈ π - π stacking with benzene ring of ACT.

B. *Neutral pH (pH =pHpzc)*:

- Surface charge is nearly neutral.
- ACT remains neutral.

Adsorption mechanisms are similar to those under acidic conditions but slightly reduced due to weaker hydrogen bonding and surface interactions, rather than the absence of electrostatic attraction.

C. *Alkaline pH (pH > pHpzc)* :

- Surface becomes negatively charged (-COO^-^, -Fe-O^-^).
- ACT becomes anionic (ACT-O^-^).

*Electrostatic Repulsion*:

-Fe-O^−^ × ACT-O^−^ → Repulsion.

Reduced hydrogen bonding and weaker van der Waals interactions lead to lower adsorption efficiency.

Weak -COO-… NH-COCH_3_.

*Surface protonation under acidic conditions (pH < pHpzc)*:

These equations summarize how surface charge and ACT ionization influence the adsorption process at various pH levels. Here is the adsorption mechanism of acetaminophen (ACT) onto Nano-iron loaded pomegranate peel powder (NIL-PP) under different pH conditions, expressed using chemical equations.

pH < 6.7 → [-Fe-OH_2_^+^] + ACT⁰ →Adsorption ↑↑↑ (via H-bond, Fe coordination).

pH ≈ 6.7 → [-Fe-OH] + ACT⁰ → Adsorption ↑↑ (moderate).

pH > 6.7 → [-Fe-O^−^] + ACT-O^−^ → Adsorption ↓↓ (repulsion).

#### Influence of drug content on the adsorption process

**Fig. 11:**
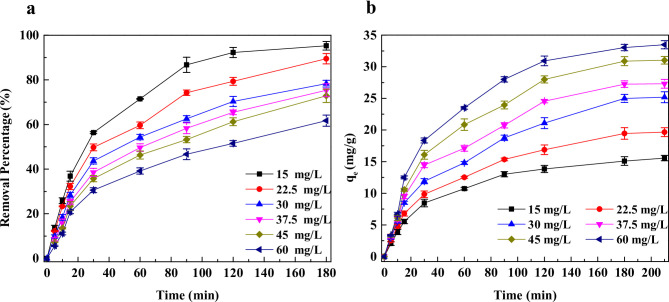
Influence of initial ACT concentration on adsorption by NIL-PP under controlled experimental conditions (adsorbent dosage: 1 g/L; solution pH = 5; temperature = 20 °C; agitation speed = 250 rpm). (**a**) Removal efficiency (%) as a function of contact time at different initial ACT concentrations (15–60 mg/L). (**b**) Adsorption capacity (qₜ, mg/g) as a function of contact time at different initial ACT concentrations (15–60 mg/L).

Figure 11a illustrates the effect of initial ACT concentration (15–60 mg/L) on the removal efficiency (%) of NIL-PP as a function of contact time. At all concentrations, adsorption proceeds rapidly during the first 20–30 min, followed by a gradual approach to equilibrium around 150–180 min. The rapid initial uptake is attributed to the abundance of readily accessible active sites and a strong concentration gradient driving mass transfer. As time progresses, the rate decreases due to progressive occupation of adsorption sites and increased diffusion resistance.

A clear inverse relationship between initial concentration and removal percentage is observed. The highest removal efficiency (≈ 95%) is achieved at 15 mg/L after 180 min, whereas the removal percentage decreases progressively with increasing concentration, reaching the lowest value at 60 mg/L. This behavior reflects surface site saturation at higher solute concentrations, where the ratio of ACT molecules to available binding sites increases, leading to competition for active sites and reduced overall percentage removal^48^.

Figure 11b, however, presents the adsorption capacity (qₜ, mg/g) versus time under the same conditions. In contrast to removal efficiency, the adsorption capacity increases with increasing initial ACT concentration. The equilibrium adsorption capacity rises from the lowest value at 15 mg/L to the highest value at 60 mg/L. This trend is expected because higher initial concentrations provide a stronger driving force for mass transfer and increase the probability of adsorbate–adsorbent interactions, allowing more ACT molecules to be loaded per unit mass of NIL-PP^50^.

Together, Fig. 11a and b demonstrate two complementary aspects of the adsorption process: while percentage removal decreases at higher concentrations due to site saturation, the adsorption capacity increases due to enhanced mass transfer driving force. The observed plateau in both plots confirms that equilibrium is reached within approximately 180 min. These findings highlight the concentration-dependent adsorption behavior of NIL-PP and emphasize the importance of optimizing operating conditions based on contaminant load for practical wastewater treatment applications.

#### Influence of adsorbent dosage on adsorption process

**Fig. 12:**
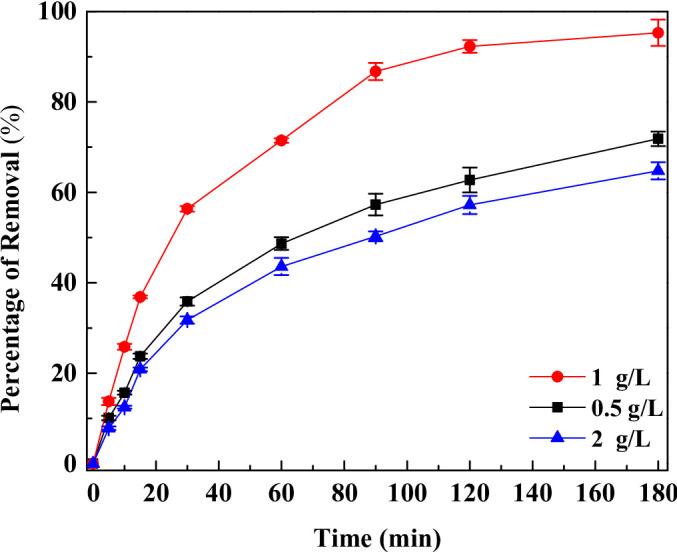
Influence of NIL-PP Dosage on ACT Adsorption under controlled experimental conditions (initial ACT concentration: 15 mg/L; solution pH: 5; temperature: 20 °C; and agitation speed: 250 rpm).

The effect of adsorbent dosage (0.5 g/L, 1 g/L, and 2 g/L) on the percentage of removal of ACT onto NIL-PP adsorbent was then investigated (Figure As shown in Fig. 12, the removal percentage was much higher when the dosage of NIL-PP was applied, and the highest removal efficiency of ∼95% was reached at 180 min with 1 g/L dosage of NIL-PP. This improvement is ascribed to the higher amount of accessible active binding sites on the NIL-PP surface, allowing adsorbate molecules to interact with the adsorbent surface^51^.

However, an increase of ACT dosage to 2 g/L significantly decreases ACT removal ability (∼ 65%). The reported phenomenon can be explained by the agglomeration of the particles at higher adsorbent dosages, which reduces the effective surface area for adsorption. In addition, the completion and overlapping of adsorption sites, and shorter diffusion path lengths may further hinder mass transfer and lower the per unit mass adsorption capacity^35^. This behavior is typical of a batch adsorption system, whereby excess adsorbent hinders the adsorption, owing to particle-particle interactions^52^.

This ideal dose (1 g/L) strikes a balance between the saturation of active sites and the dispersion of particles in solution, which favours high adsorption kinetics. Exceeding this dosage results in no further or even reduced efficiencies of removal due to either saturation of the solution with active sites or that of competing particles with active sites. Such results stress the need for proper optimization of adsorbent dose to achieve maximum efficiency at minimum cost in water technologies using biomass-based composites like NIL-PP.

Overall, all NIL-PP dosages significantly affect the adsorption property of NIL-PP for ACT removal. More dosage comes with more adsorption sites, but at a high dosage, aggregate blocking reaction sites limit the overall efficiency of the reaction. This highlighted that the adsorption experiment should be scaled to the production for practical application and economy, which is why the optimal dose of adsorbent needs to be determined. These outcomes are in accordance with the previous reports of a similar type of adsorbent derived from a biosorbent impregnated with iron for the removal of pharmaceuticals^53^.

#### Influence of temperature on the adsorption process

**Fig. 13:**
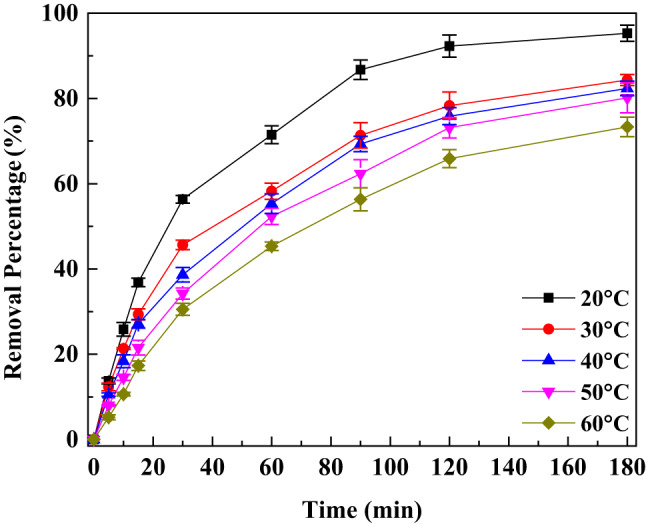
Influence of Temperature on ACT Adsorption onto NIL-PP under controlled experimental conditions (initial ACT concentration: 15 mg/L; adsorbent dosage: 1 g/L; solution pH: 5; and agitation speed: 250 rpm).

The results clearly demonstrate a pronounced influence of temperature on the adsorption performance of ACT onto NIL-PP. The removal efficiency progressively decreased with increasing temperature, reaching its highest value (≈ 95%) at 20 °C, which indicates that the adsorption process is exothermic in nature^21^. The enhanced performance at lower temperatures suggests stronger adsorbate–adsorbent interactions and greater stability of ACT molecules on the active adsorption sites^50^.

At elevated temperatures (50–60 °C), the removal efficiency exhibited a slight decline or plateau, which may be attributed to the increased kinetic energy of ACT molecules that promotes desorption and weakens the intermolecular forces governing adsorption. Excessive thermal agitation likely disrupts the weak physicochemical interactions, such as hydrogen bonding and van der Waals forces, thereby reducing the net adsorption capacity (Fig. 13).

Although moderate temperature increases may facilitate molecular diffusion and intraparticle transport within the NIL-PP pores, the overall decrease in adsorption efficiency confirms that the thermodynamic driving force favors adsorption at lower temperatures. These findings support a predominantly physisorption-controlled and exothermic mechanism. Consequently, operation at ambient or slightly lower temperatures is recommended to maximize removal efficiency, offering practical advantages for real wastewater treatment applications where energy-efficient conditions are desirable^48^.

#### Impact of particle size on the process of adsorption

**Fig. 14:**
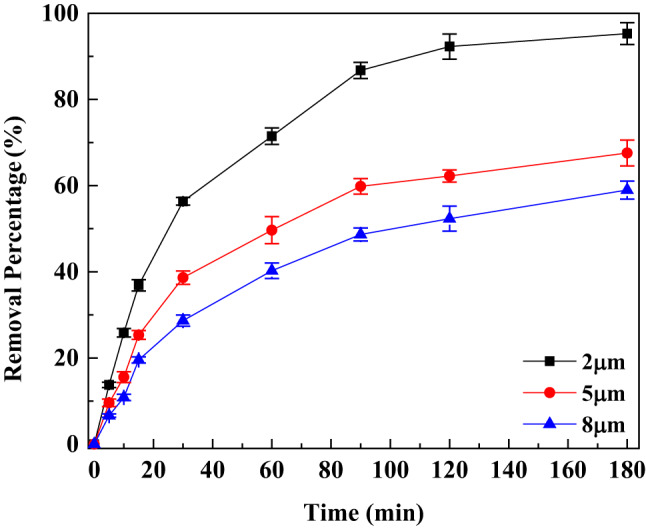
Effect of particle size on ACT adsorption using NIL-PP under controlled experimental conditions (initial ACT concentration: 15 mg/L; adsorbent dosage: 1 g/L; solution pH: 5; temperature: 20 °C; and agitation speed: 250 rpm).

The effect of adsorbent particle size on the adsorption of ACT onto NIL-PP was investigated. As shown in Fig. 14, the removal efficiency increased with decreasing particle size over time. The smallest particle size (2 μm) exhibited the highest removal efficiency, reaching approximately 95% after 180 min, whereas larger particles (5 μm and 8 μm) showed significantly lower removal efficiencies throughout the adsorption period. This behavior can be attributed to the higher surface area-to-volume ratio of smaller particles, which provides a greater number of accessible adsorption sites for ACT molecules, thereby enhancing adsorption performance. The particle size values reported correspond to the effective particle dimensions after processing and dispersion and should not be interpreted as sieve aperture sizes^54^.

In addition, smaller particle sizes facilitate the diffusion of ACT molecules to active sites, thereby reducing mas transfer resistance^55^. The 2 μm NIL-PP particles possess higher surface reactivity and larger external surface area, resulting in faster adsorption kinetics and improved adsorption performance. In contrast, larger particles offer fewer accessible surface sites and longer internal diffusion pathways, which can slow down the adsorption rate. Moreover, smaller NIL-PP particles exhibit improved dispersion and pore accessibility in aqueous media, allowing ACT molecules to interact more effectively with iron-loaded functional groups. For larger particles, reduced adsorption efficiency may be associated with internal diffusion limitations or partial pore blockage, hindering access to internal adsorption sites^3^. Overall, particle size is a crucial factor influencing the adsorption performance of NIL-PP, and smaller particle sizes enhance both adsorption efficiency and kinetics, providing valuable insight for the practical design of efficient adsorption systems.

#### Influence of stirring speed on the adsorption process

**Fig. 15:**
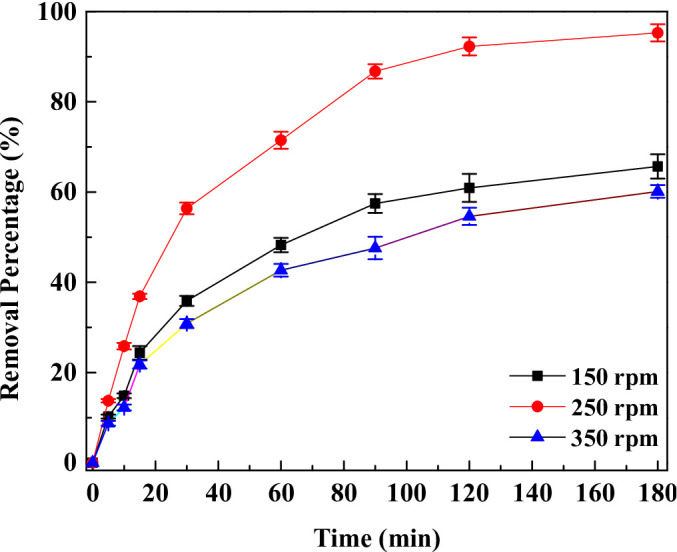
Influence of stirring speed on ACT Adsorption onto NIL-PP under controlled experimental conditions (initial ACT concentration: 15 mg/L; adsorbent dosage: 1 g/L; solution pH: 5; and temperature: 20 °C).

The adsorption of ACT onto NIL-PP is affected distinctly by the stirring speed. As observed from Fig. 15, the removal percentage of ACT increased with stirring speed when changing stirring speed from 150 rpm to 250 rpm, and the percentage reached ∼95% after 180 min. However, the adsorption efficiency decreased at 350 rpm. At greater agitation rates, the pattern can be explained by the balance between the mass transfer enhancement effect and the desorption of particles^56^.

However, the slow stirring rate (250 rpm) does seem to affect the adsorption kinetics trends, as agitating the solution would disperse the adsorbent particles homogeneously throughout the solution and decrease the mass transfer resistance around each particle, which is determined by the thickness of the boundary layer, and hence increase the mass transfer rate of ACT molecules to active adsorption sites. This enhanced mixing efficiency results in increased surface contact and diffusion, which accounts for the higher performance at this speed^57^.

In contrast, at higher stirring speeds (350 rpm), the lower removal efficiency might be attributed to desorption of weakly bound ACT molecules caused by the turbulence or possible disruption of the adsorbent skeleton. Overly aggressive stirring can also contribute to clump particles, reducing the surface area and effective adsorption capacity^57^. This finding suggests that the stirring speed can be used to achieve a balance between the constant stirring to promote adsorption and the risk of dislodging more fragile structures of the adsorbent.

The data highlight an ideal stirring speed (250 rpm) at which mass transport is maximised, without adversely affecting particle dynamics. It is consistent with other studies indicating that the optimal operational parameters must be determined to achieve efficient removal of contaminants from the aqueous phase using bio-based nanocomposite adsorbents^56^.

#### Reusability study

**Fig. 16:**
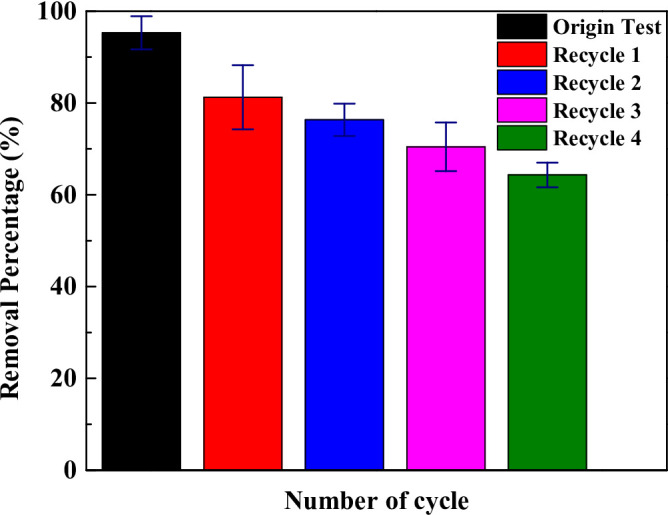
Recyclability of NIL-PP as a function of ACT sdsorption under controlled experimental conditions (initial ACT concentration: 15 mg/L; adsorbent dosage: 1 g/L; solution pH: 5; temperature: 20 °C; and agitation speed: 250 rpm).

Recyclability of adsorbent is an important factor in their applicability for wastewater treatment NIL-PP is gradual decline in ACT removal efficiency with reuse cycles (as shown in Fig. 16). In the first cycle of the original experiment, the removal efficiency was 95.29%, but this value decreased to 81.24%, 76.33%, 70.45%, and 64.33% during subsequent cycles. The retained efficiency was substantial up to four cycles. The retained efficiency was substantial up to four cycles (efficiency still > 64%) as indicated in Fig. 16, which reflected great reusability of NIL-PP despite the observed decrease.

The reduction in adsorption ability is basically attributed to partial loss of the active sites or blockage of the pores, structural disintegration of the adsorbent, or incomplete desorption of acetaminophen after every regeneration step. These effects decrease the accessibility of surface functional groups (e.g., hydroxyl, carboxyl, and iron oxide moieties) that are mainly associated with hydrogen bonding and specific surface interactions with ACT molecules, while electrostatic interactions, if present, play only a secondary role under the studied pH conditions^59^.

However, the small reduction in performance suggests that NIL-PP has potential as a bio-based material with economic and environmental sustainability for several use cycles. Regeneration processes like washing the solvent, pretreating with a mild acid/base, or microwave regeneration can make this adsorbent more sustainable and longer-living than the rest^60^. Retention of removal capacity > 60% after four cycles is in accordance with trends associated with similar biosorbents produced from agricultural waste. Although regeneration experiments demonstrated that NIL-PP retained a substantial fraction of its adsorption capacity after reuse, detailed post-regeneration structural characterization (e.g., FTIR, XRD, or SEM) was not conducted. Therefore, the structural stability of NIL-PP is inferred indirectly from its sustained adsorption performance. Nevertheless, NIL-PP exhibited an acceptable degree of reusability, indicating its potential as a cost-effective and sustainable adsorbent for pharmaceutical-contaminated wastewaters requiring multiple treatment cycles. Further studies focusing on optimized regeneration protocols and post-regeneration structural analyses are recommended to enhance its regenerative efficiency and assess its long-term applicability in large-scale systems.

#### Thermodynamic analysis of ACT adsorption onto NIL-PP

The thermodynamic behavior of acetaminophen (ACT) adsorption onto NIL-PP was evaluated over the temperature range of 20–60 °C to elucidate the feasibility and nature of the adsorption process. The thermodynamic relationships are illustrated in Fig. 13a and b, while the calculated parameters are summarized in Table 4.

The standard Gibbs free energy change (ΔG⁰) was estimated using the Van’t Hoff equation based on the Langmuir equilibrium constant (K_L_):

3 $$\Delta G^{\circ}= {\text{ }} - {\text{RTln }}\left( {{\mathrm{K}}_{{\mathrm{L}}} } \right)$$

where R is the universal gas constant (0.008314 kJ/mol K) and T is the absolute temperature (K). The temperature dependence of the standard Gibbs free energy change (ΔG⁰) follows the Gibbs–Helmholtz relationship, which relates ΔG⁰ to the corresponding changes in enthalpy (ΔH⁰) and entropy (ΔS⁰) according to:

4 $$\Delta G^{\circ}=\Delta H^{\circ}-T\Delta S^{\circ}$$

Figure 13a presents the Van’t Hoff plot (ln K_L_ versus 1/T), which was used to determine the standard enthalpy (ΔH⁰) and entropy (ΔS⁰) changes, as reported in Table 4. The negative enthalpy change (ΔH⁰ = −47.13 kJ/mol), derived from the slope of the Van’t Hoff plot, confirms the exothermic nature of ACT adsorption onto NIL-PP^60^ in contrast, the positive entropy change (ΔS⁰ = +0.198 kJ/mol K) indicates an increase in randomness at the solid–liquid interface during the adsorption process^61,62^.

Figure 13b illustrates the variation of ΔG⁰ with temperature, with the corresponding numerical values listed in Table 4. The negative ΔG⁰ values at all investigated temperatures confirm that ACT adsorption onto NIL-PP is spontaneous. However, the gradual decrease in the magnitude of ΔG⁰ with increasing temperature indicates reduced adsorption favorability at elevated temperatures, which is characteristic of exothermic adsorption systems^30^.

As a result, the positive ∆S⁰ values indicate an increase in randomness at the solid–liquid interface following ACT adsorption, which can be attributed to the displacement of water molecules and the formation of a more dynamically favorable arrangement of ACT molecules on the NIL-PP binding sites^59,63^. Similar thermodynamic behavior has been reported in previous studies on bio-waste-derived adsorbents for pharmaceutical removal, including modified biochars and iron-doped biosorbents^64^.

**Fig. 17:**
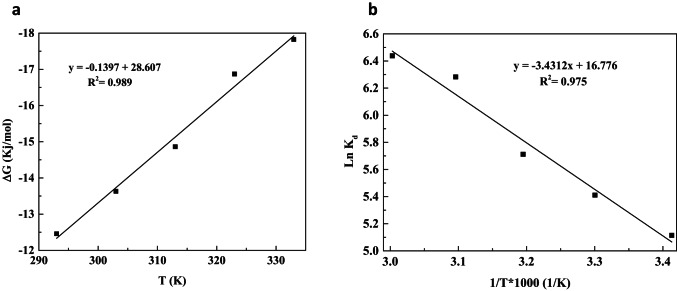
(**a** & **b**) Thermodynamics Parameter.

**Fig. 18:**
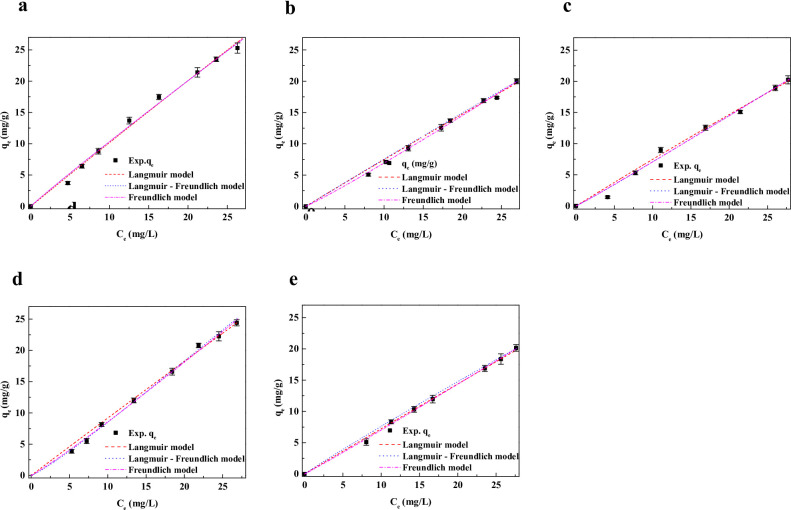
Freundlich and Langmuir-Freundlich, and Langmuir model isotherm plots for Paracetamol adsorption on NIL-PP at different temperatures (**a**) 20, (**b**) 30, (**c**) 40, (**d**) 50, and (**e**) 60 °C. Under controlled experimental conditions (initial ACT concentration: 15 mg/L; adsorbent dosage: 1 g/L; solution pH: 5; and agitation speed: 250 rpm).

The thermodynamic evaluation demonstrates that ACT adsorption onto NIL-PP is spontaneous and exothermic, accompanied by increased interfacial disorder. These findings reinforce the suitability of NIL-PP as an efficient biosorbent under ambient conditions, while indicating that operation at elevated temperatures may require further optimization due to weakened adsorbate–adsorbent interactions (Figs. 17,18).

**Table 4:** Thermodynamic parameters.

T (K)	KL (L/mg)	ΔG° (kJ/mol)	ΔH (kJ/mol)	ΔS (kJ/mol.K)
**293**	0.0011	−12.46	−47.13	0.198
**303**	0.0015	−13.63		
**313**	0.002	−14.86		
**323**	0.0035	−16.87		
**333**	0.0041	−17.82		

#### Adsorption isotherms

Adsorption isotherms are essential for defining the interaction between adsorbent and adsorbate, and predicting the equilibrium adsorption capacity of materials^21^. Therefore, the present work investigates the equilibrium adsorption behavior of acetaminophen (ACT) on nano-iron-loaded pomegranate peel powder (NIL-PP) over a wide range of temperatures (i.e., at 20 °C, 30 °C, 40 °C, 50 °C, and 60 °C). To fit the experimental data, three nonlinear isotherm models, namely Freundlich (Eq. 6), Langmuir, and Langmuir-Freundlich model (Eq. 7) were used^43,44^. They provide different mechanistic insights whereby Langmuir assumes monolayer adsorption to a homogenous surface^21^, Freundlich considers multilayer adsorption to a heterogeneous surface, and the Langmuir-Freundlich model incorporates both behaviors, which is particularly useful for complex surfaces, including biomass-based adsorbents.

5 $${\mathrm{Q}} = {\mathrm{K}}_{{\mathrm{f}}} {\mathrm{C}}_{{\mathrm{e}}} ^{{{\mathrm{1}}/{\mathrm{n}}}}$$

6 $${\mathrm{q}} = {\text{ K}}_{{\mathrm{L}}} {\mathrm{q}}_{{\mathrm{m}}} {\mathrm{Ce}}^{{{\mathrm{1}}/{\mathrm{n}}}} \left( {{\mathrm{1}} + {\mathrm{K}}_{{\mathrm{L}}} {\mathrm{Ce}}^{{{\mathrm{1}}/{\mathrm{n}}}} } \right)$$

7 $${\mathrm{q}} = {\text{ K}}_{{\mathrm{L}}} {\mathrm{q}}_{{\mathrm{m}}} {\mathrm{C}}_{{\mathrm{e}}} ^{{{\mathrm{1}}/{\mathrm{n}}}} /\left( {{\mathrm{1}} + {\mathrm{K}}_{{\mathrm{L}}} {\mathrm{Ce}}^{{{\mathrm{1}}/{\mathrm{n}}}} } \right)$$

where q is the amount of adsorbed ACT at equilibrium settings (mg/g), C_e_ is the ACT equilibrium concentration in solution mg/L), q_m_ is the adsorbent’s maximum adsorption capacity (mg/g), K_L_ is the Langmuir constant related to the adsorption energy (L/mg), and KL and n are the Freundlich adsorption constants.

Fitting models using nonlinear regression through Excel Solver demonstrated inconsistent performance between temperature regimes. Langmuir and Langmuir-Freundlich models provided strong fits to the experimental data, with high R^2^ values and low error indicators (RMSE and χ^2^) at lower temperatures (20–30 °C), indicating a predominant monolayer adsorption process with constant energy distribution^65^. However, at higher temperatures, the data fitted well to the Freundlich and Langmuir-Freundlich equations, suggesting greater surface heterogeneity and multilayered adsorption^65^. This transition emphasizes surface property changes driven by temperature, possibly due to altered porosity and accessibility to binding sites, similar to recent temperature-dependent pharmaceutical adsorption studies.

The maximum monolayer adsorption capacity (qₘ) obtained from the Langmuir and Langmuir–Freundlich models decreased significantly as the temperature increased from 20 to 60 °C (i.e., 944.4 mg/g to 217.8 mg/g (Langmuir) and 1090 mg/g to 228.9 mg/g (Langmuir- Freundlich), This pronounced decrease confirms the exothermic nature of the adsorption process, as increased thermal energy weakens the adsorbent–adsorbate interactions, likely disrupting hydrogen bonding and electron donor–acceptor interactions between ACT molecules and the functional groups of NIL-PP^66^. The high Langmuir and Langmuir-Feundlich adsorption capacity reflect the strong affinity and site-specific interactions between ACT and NIL-PP rather than simple physical adsorption governed by surface area. Moreover, the higher qₘ values at lower temperatures indicate that NIL-PP is more suitable for adsorption under ambient temperature conditions.

While commercial activated carbon is a benchmark adsorbent with high surface area and strong adsorption capacities, it is relatively expensive due to energy-intensive production processes. In contrast, low-cost biosorbents and biochar-based materials such as NIL-PP can achieve comparable adsorption performance for selected pharmaceuticals and organic contaminants at substantially lower material cost, owing to the use of abundant agricultural waste feedstock and simpler preparation methods. For example, modified activated carbons derived from pomegranate peel have been reported to exhibit adsorption capacities of several hundred mg/g for organic pollutants, and iron-modified biosorbents commonly demonstrate enhanced uptake compared with their unmodified counterparts^52^. These observations highlight the competitive potential of NIL-PP as an economical and effective adsorbent for practical water treatment applications^67^.

The Freundlich constant (n) provides insight into adsorption intensity and surface heterogeneity. In the present study, the n values obtained from both the Freundlich and Langmuir–Freundlich models ranged from 0.830 to 1.044, indicating nearly linear adsorption behavior with increasing surface heterogeneity at higher temperatures. The gradual decrease in n with increasing temperature reflects a weakening of adsorbate–adsorbent interactions due to thermal agitation, supporting the exothermic nature of the adsorption process. Similar trends have been reported for the adsorption of pharmaceutical compounds on biochar-based and iron-impregnated biosorbents, where stronger binding occurs at lower temperatures^68^.

The dimensionless separation factor (RL) is a characteristic parameter used to describe the adsorption process, and it was determined based on the Langmuir relationship, where R_L_ values between 0 and 1 indicate that ACT adsorption on NIL-PP was favorable at all temperatures under study^21^. Systematic model comparison via standard error (S.E.), chi-square (χ^2^), and RMSE also validated referential predictions from the Freundlich and Langmuir-Freundlich model and confirmed higher predictive accuracy than for the simpler two-parameter Langmuir model. The distribution of sites on the NIL-PP surface indicates the complexity of the NIL-PP that remains after iron nanoparticles are dispersed in a heterogeneous lignocellulosic matrix^21^.

8 $$\:{R}_{L}=\frac{1}{{1+K}_{L}{C}_{O}}$$

Where “C_o_” is the starting concentration and ‘K_L_’ is the Langmuir constant. The ACT is more favorably adsorbed on the NIL-PP Nano-composite (Table 5).

The adsorption of ACT on the NIL-PP is fitted well by the Langmuir-Freundlich and the Freundlich models, especially at the studied higher temperatures, suggesting the adsorption process changed from monolayer to multilayer with increased surface heterogeneity. The reduction in adsorption capacity with increasing temperature indicates the exothermic nature of the process, favoring adsorption at ambient conditions. These investigations confirm that NIL-PP can be considered as a sustainable solid phase adsorbent for the removal of pharmaceuticals, particularly in decentralized wastewater treatment systems applied at room-temperature conditions. Further work should be devoted to the possibility of regeneration of competitive adsorption in multicomponent systems to make practical use of advanced^49^.

**Table 5:** Estimated isotherm parameters for Paracetamol adsorption using NIL-PP as adsorbent at different constant temperature.

	Adsorption 20 °C	Adsorption 30 °C	Adsorption 40 °C	Adsorption 50 °C	Adsorption 60 °C
q = K_f_ C_e_^1/*n*^	Freundlich isotherm model				
K_f_	1.139	0.594	0.658	0.717	0.444
N	1.044	0.977	0.969	0.928	0.830
R^2^	0.991	0.996	0.990	0.996	0.958
SE	0.847	0.357	0.782	0.532	1.806
RMSE	0.785	0.330	0.724	0.493	1.672
χ^2^	0.518	0.872	0.905	0.165	2.177
q= K_L_q_m_C_e_/(1 + K_L_C_e_)	Langmuir isotherm model				
K_L_ (L/mg)	0.0011	0.0015	0.002	0.0035	0.004
qm (mg/L)	944.4	520.7	381.5	276.4	217.8
R^2^	0.991	0.997	0.989	0.988	0.928
SE	0.845	0.325	0.834	0.939	2.375
RMSE	0.782	0.274	0.772	0.869	2.199
χ^2^	0.441	0.064	1.072	0.699	4. 172
R_L1_	0.984	0.981	0.935	0.982	0.984
q=K_L_q_m_C_e_^1/n^/(1 + K_L_Ce^1/n^)	Langmuir–Freundlich isotherm model				
K_L_ (L/mg)^1/n^	0.001	0.0014	0.0015	0.0021	0.0016
q_m_ (mg/L)	1090	559.2	412.9	297.6	228.9
N	1.029	0.997	0.94	0.865	0.778
R^2^	0.991	0.991	0.990	0.996	0.961
SE	0.908	0.654	0.839	0.523	1.921
RMSE	0.767	0.553	0.709	0.434	1.623
χ2	0.497	0.263	0.864	0.103	2.010
R_L2_	0.986	0.954	0.976	0.965	0.968

### Kinetic and intraparticle diffusion modelling for ACT adsorption onto NIL-PP

**Fig. 19:**
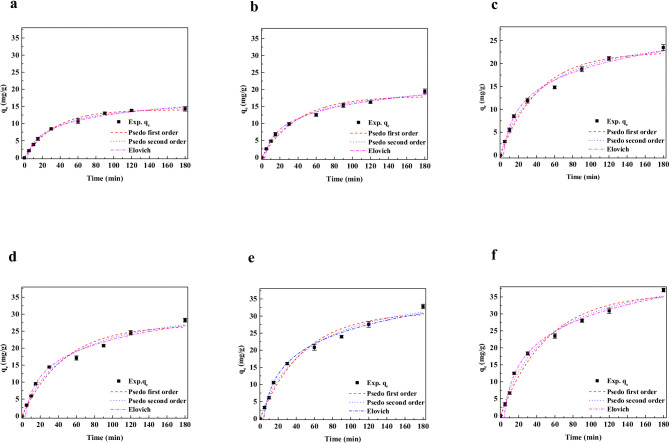
Psedo-first order, Psedo-second order, Elovich model Kinetics plots at different concentration of Paracetamol (**a**) 15, (**b**) 22.5, (**c**) 30, (**d**) 37.5, (**e**) 45 and (**f**) 60 mg/L using NIL-PP. Under controlled experimental conditions (adsorbent dosage: 1 g/L; solution pH: 5; temperature: 20 °C; and agitation speed: 250 rpm).

Understanding the mechanisms and driving forces governing the transfer of solute molecules from the bulk liquid phase to the adsorbent surface makes kinetic modelling a crucial aspect of adsorption studies^66^. In this work, the adsorption kinetics of acetaminophen (ACT) onto NIL-PP were investigated using the pseudo-first-order (PFO), pseudo-second-order (PSO), and Elovich models. These kinetic models provide insight into whether the adsorption process is primarily controlled by physisorption or chemisorption and reflect the energetic characteristics and heterogeneity of the adsorbent surface. The nonlinear kinetic equations applied in this study are expressed as follows: the PFO model (Eq. 9), the PSO model (Eq. 10), and the Elovich model (Eq. 11), where qt and qe (mg/g) represent the adsorption capacities at time t (h) and at equilibrium, respectively, and k₁ (h⁻¹) and k₂ (g/mg h) denote the corresponding rate constants.

The nonlinear PFO model is given by:

9 $$\:{q}_{t}={q}_{e}(1-{e}^{-k1t})$$

The nonlinear PSO model is expressed as:

10 $$\:{q}_{t}=\frac{{k}_{2}{q}_{e}^{2}t}{1+{q}_{e}{k}_{2}t}$$

The Elovich kinetic equation is written as:

11 $$\:{q}_{t}=(\frac{1}{\beta\:}ln\alpha\:\beta\:+\frac{1}{\beta\:})lnt$$

Where α (mg/g.h) represents the initial adsorption rate and β (g/mg) is related to the surface coverage and activation energy of chemisorption. The estimated kinetic parameters are summarized in Table 6, and the corresponding nonlinear fitting curves are illustrated in Fig. 19. As shown in Table 6, the pseudo-second-order model exhibits the highest correlation coefficients (R²) and the lowest error values over the investigated ACT concentration range (15–60 mg/L), together with excellent agreement between calculated (qₑ,cal) and experimental (qₑ,exp) adsorption capacities. Although the PFO model also demonstrates relatively high R² values, noticeable deviations in q_e_ estimation indicate that first-order kinetics cannot fully describe the adsorption mechanism. The Elovich model shows good correlation (R² = 0.981–0.991), supporting the heterogeneous nature of the NIL-PP surface; however, its overall statistical performance remains inferior to that of the PSO model.

The superior applicability of the PSO model, as summarized in Table 6 and confirmed in Fig. 19, indicates that ACT adsorption onto NIL-PP is predominantly governed by chemisorption^21^. The observed decrease in the PSO rate constant (k₂) with increasing initial ACT concentration suggests progressive occupation of high-energy active sites and reduced availability of chemically reactive centers at higher solute loadings^69^. Furthermore, the Elovich parameters reveal an increase in the initial adsorption rate (α) with increasing ACT concentration and a gradual decrease in β, indicating heterogeneous surface coverage and variation in adsorption energy. These findings confirm the presence of energetically non-uniform adsorption sites, likely associated with oxygen-containing functional groups and iron-modified active centers on the NIL-PP surface^70^.

To further elucidate the diffusion mechanism, the Weber–Morris intraparticle diffusion model (Eq. 12) was applied:

12 $$\:{\mathrm{q}}_{\mathrm{t}}\mathrm{=}{\mathrm{k}}_{\mathrm{p}}{\mathrm{t}}^{\mathrm{1/2}}\mathrm{+}\mathrm{C}$$

**Fig. 20:**
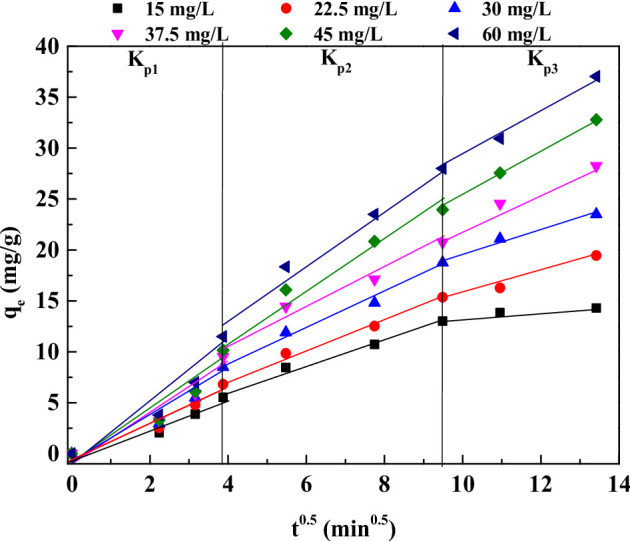
Intraparticle Diffusion Model for ACT adsorption onto NIL-PP.

Where k_p_ (mg/g.min^0.5^) is the intraparticle diffusion rate constant and C is the boundary layer’s constant thickness. The calculated parameters are presented in Table 7, and the corresponding qt versus t^0.5^ plots are shown in Fig. 20. The multilinear nature of these plots confirms that ACT adsorption proceeds through multiple stages. The initial sharp region corresponds to external film diffusion, where ACT molecules rapidly migrate from the aqueous phase to the outer surface of NIL-PP under a strong concentration gradient^71^. The second linear region is attributed to intraparticle diffusion, representing the gradual penetration of ACT molecules into the internal pores and their transport to internal binding sites^72^. The final plateau region corresponds to equilibrium attainment. Importantly, the diffusion lines do not pass through the origin (C ≠ 0), indicating that intraparticle diffusion is not the sole rate-limiting step^34^.

Overall, the combined kinetic and diffusion analyses (Tables 6 and 7; Figs. 19 and 20) demonstrate that ACT adsorption onto NIL-PP follows pseudo-second-order kinetics and is primarily controlled by chemisorption. The dominant mechanism involves strong chemical interactions between ACT functional groups (–OH, –NH, and amide moieties) and the oxygen- and iron-containing active sites on the NIL-PP surface, leading to surface complexation and monolayer formation. Although external film diffusion and intraparticle diffusion contribute to the overall adsorption pathway, the surface chemical reaction represents the principal rate-controlling step.These outcomes are in accordance with^29^.

**Table 6:** Estimated kinetic model parameters for ACT adsorption using NIL-PP at different concentrations.

ACT Concentration →	15 mg/L	22.5 mg/L	30 mg/L	37.5 mg/L	45 mg/L	60 mg/L
q_t_=k_2_q_e_^2^t/(1 + q_e_k_2_t)						
*q_e_	14.04	18.02	22.50	26.77	31.10	36.09
k_2_	0.029	0.025	0.023	0.021	0.020	0.019
R^2^	0.997	0.991	0.991	0.986	0.991	0.990
SE	0.299	0.659	0.842	1.258	1.194	1.394
NSD	1.643	1.797	1.543	1.199	0.797	3.081
ARE	1.438	1.572	1.350	1.049	0.698	2.695
q = qe(1-exp(-k_1_t))						
q_e_	17.28	22.53	28.26	33.91	39.93	44.95
k_1_	0.0018	0.001	0.0008	0.0006	0.0005	0.0004
R^2^	0.994	0.976	0.978	0.971	0.979	0.978
SE	0.449	1.099	1.311	1.787	1.768	2.044
NSD	2.519	6.507	6.566	6.512	4.297	4.794
ARE	2.203	5.295	5.745	5.476	3.760	4.195
**q_t_=(1/β)ln(αβ) + 1/β)lnt						
Β	0.271	0.215	0.173	0.145	0.122	0.108
Α	1.179	1.376	1.636	1.824	1.995	2.258
R^2^	0.991	0.990	0.987	0.981	0.986	0.989
SE	0.474	0.640	0.909	1.321	0.018	0.200
NSD	0.561	2.157	2.368	2.805	3.499	3.145
ARE	0.490	1.887	2.072	2.454	3.062	2.752

*q; adsorbed As (mg/g) at time t (h).

** The simple Elovich parameters were estimated without using the origin (q = 0, t = 0).

**Table 7:** Intra-particle diffusion model for ACT adsorption using NIL-PP at different concentrations.

Linear portion	Constant	15 mg/l	22.5 mg/l	30 mg/l	37.5 mg/l	45 mg/l	60 mg/l
First	KP_1_ (mg/g min^0.5^)	1.170	1.447	1.663	1.787	1.842	2.116
	C_1_ (mg/g)	0.126	0.160	0.173	0.177	0.190	0.211
	R^2^	0.961	0.960	0.964	0.967	0.964	0.966
Second	KP_2_ (mg/g min^0.5^)	1.318	1.456	1. 604	1.912	2.718	3.034
	C_2_ (mg/g)	0.726	1.428	2.593	2.795	0.209	0.443
	R^2^	0.971	0.982	0.980	0.929	0.974	0.968
Third	KP_3_ (mg/g min^0.5^)	0.312	1.069	1.181	1.868	4.414	2.317
	C_3_ (mg/g)	10.20	4.961	7.790	3.430	19.27	5.838
	R^2^	0.905	0.974	0.981	0.977	0.989	0.997

## Conclusions

The prepared Nano Iron Loaded on pomegranate peel (NIL-PP) is a promising, magnetically recoverable biosorbent for ACT removal. The comprehensive characterization confirmed nanoscale iron specie anchored on a porous biochar matrix, exhibiting a sufficient magnetic response (Ms ≈ 18.5 emu/g). The batch adsorption experiments demonstrated that the process follows pseudo-second-order kinetics and is best described by the Freundlich isotherm model, indicating that adsorption occurs predominantly through chemisorption on energetically heterogeneous surfaces with multilayer coverage. Optimal performance was achieved at an adsorbent dose of 1 g/L, under slightly acidic conditions pH 5, an initial ACT concentration of 15 mg/L, and ambient temperature. Thermodynamic analysis showed the adsorption to be exothermic, with explains the decline in removal efficiency at higher temperatures. The removal mechanism likely involves a combination of pore filling, hydrogen bonding and van der waals interactions with oxygenated surface groups, π–π interactions with the aromatic rings of ACT, and surface complexaiton at iron sites. The iron phase not only increases the density of active sites but also enable facile magnetic separation. NIL-PP maintained a considerable fraction of its adsorption capacity after reuse (64% after four cycles). However, further evaluation under real and complex wastewater conditions, together with synthesis optimization and cost analysis aimed at enhancing adsorption capacity while minimizing secondary contamination, is required. Future studies will focus on detailed structural and morphological analyses after repeated regeneration cycles to fully assess the long-term stability and reusability of NIL-PP. Addressing these aspects will help establish the environmental safety, economic feasibility, and scalability of NIL-PP as a low-cost treatment option for pharmaceutical-contaminated waters.

## Data Availability

Data are available on request from the authors.
